# Polymer Composites in Additive Manufacturing: Current Technologies, Applications, and Emerging Trends

**DOI:** 10.3390/polym18020192

**Published:** 2026-01-10

**Authors:** Md Mahbubur Rahman, Safkat Islam, Mubasshira, Md Shaiful Islam, Raju Ahammad, Md Ashraful Islam, Md Abdul Hasib, Md Shohanur Rahman, Raza Moshwan, M. Monjurul Ehsan, Md Sanaul Rabbi, Md Moniruzzaman, Muhammad Altaf Nazir, Wei-Di Liu

**Affiliations:** 1 Department of Mechanical Engineering, Khulna University of Engineering & Technology, Khulna 9203, Bangladesh; safkat.me2k17kuet@gmail.com (S.I.); shaifulislam.me16@gmail.com (M.S.I.); rajuahammad@me.kuet.ac.bd (R.A.); md.islam@me.kuet.ac.bd (M.A.I.); ahasib@me.kuet.ac.bd (M.A.H.); 2 Department of Material Science and Engineering, Khulna University of Engineering & Technology, Khulna 9203, Bangladesh; mubasshira1927020@stud.kuet.ac.bd; 3 Department of Mechatronics Engineering, Khulna University of Engineering & Technology, Khulna 9203, Bangladesh; shohan@mte.kuet.ac.bd; 4 Department of Mechanical Engineering, IUBAT—International University of Business Agriculture and Technology, Dhaka 1230, Bangladesh; moshwan.me@iubat.edu; 5 Department of Mechanical and Production Engineering (MPE), Islamic University of Technology (IUT), Board Bazaar, Gazipur 1704, Bangladesh; ehsan@iut-dhaka.edu; 6 Department of Mechanical Engineering, Chittagong University of Engineering and Technology, Chattogram 4349, Bangladesh; rabbi@cuet.ac.bd; 7 Department of Textile Engineering, Khulna University of Engineering & Technology, Khulna 9203, Bangladesh; md.moniruzzaman@usm.edu; 8 School of Polymer Science and Engineering, The University of Southern Mississippi, Hattiesburg, MS 39406, USA; 9 Institute of Chemistry, The Islamia University of Bahawalpur, Bahawalpur 63100, Pakistan; altaf.nazir@iub.edu.pk; 10 Herbert Gleiter Institute of Nanoscience, School of Materials Science and Engineering, Nanjing University of Science and Technology, Nanjing 210094, China; weidi.liu@njust.edu.cn

**Keywords:** polymer composites, additive manufacturing, fiber-reinforced polymers, multi-material printing, in situ curing

## Abstract

Polymer composites have opened a novel innovation phase in additive manufacturing (AM), and now lightweight, high-strength, and geometrical advanced components with tailored functionalities can be produced. The present study introduces advances in polymer composite materials and their integration into AM processes, particularly in rapidly growing industries such as aerospace, automotive, biomedical, and electronics. The embedding of cutting-edge reinforcement materials, such as nanoparticles, carbon fibers, and natural fibers, into polymer matrices enhances mechanical, thermal, electrical, and multifunctional properties. These material developments are combined with advanced fabrication techniques, including multi-material printing, in situ curing, and functionally graded manufacturing, which achieves accurate regulation of microstructures and properties. Furthermore, high-impact innovations such as smart polymer composites with self-healing or stimuli-responsive behaviors, the growing shift toward sustainable, bio-based composite alternatives, are driving progress. Despite significant advances, challenges remain in interfacial bonding, printability, process repeatability, and long-term durability. This review offers a comprehensive overview of current advancements and outlines future directions in polymer composite–based AM.

## 1. Introduction

Additive manufacturing (AM), also known as 3D printing, is a game-changing manufacturing concept that is changing the design, materials engineering, and manufacturing processes in numerous industries. In contrast to traditional subtractive or mold-based production methods, AM allows direct digital-to-physical printing with less material waste, less tooling demands, and more design flexibility; thus, increasing economic and production efficiency [1]. Among the greatest benefits of AM is the absence of tooling in the form of molds, which contributes to quick prototyping and the production of personalized parts. Computer-aided design (CAD) is therefore at the core of AM since it allows optimization of geometry, controlled delivery of materials to the physical structure, and fluid transfer of digital models to the real world [2]. Though AM research has been developed actively together with digital design and computational tools, the low printability and lack of mechanical performance of monolithic polymers have limited the functionality of these materials for useful and load-bearing purposes. This shortcoming has led to intensive studies on customized polymer blends and polymer-based composites, which are associated with better mechanical stability, material efficiency, and performance, as well as less waste and a lower cost of production [3]. Long-fiber reinforced composites and especially synthetic fiber composites like glass-reinforced plastics (GRP) were initially developed in the middle of the last century, and since that time, manufacturing strategies have developed in order to improve the performance of structures and increase productivity [4]. With this combination of composite systems with AM today, it is possible to create complex architectures that are hard or impossible to create through other traditional molding or lamination methods. The potential of polymer-based AM has been significant in the engineering fields, particularly because it can produce composites with engineered material profiles [5]. Polymer-based AM methods have been used in a wide range of engineering industries in fabricating composites with engineered material profiles. However, difficulties are faced during the choice of their phase distributions, fiber aspect ratio, and the short or continuous fiber arrangement. These elements have an effect on the feedstock and printing strategy design [6]. Surface finish and resolution, as well as other similar constraints in AM, can be technique-dependent and might often be evaluated on a context-dependent basis. In early AM, the achievement of macro-scale geometric complexity was the challenge, and modern developments in voxel- and path-control of materials can be obtained using droplet-based printing, which allows local functionality and spatially programmed behavior [7].

AM has now emerged as a prospective technology in the automobile industry using polymers, which could enable a lighter structure, increased design flexibility, and new composite applications. It approves light constructions, useful design, and innovativeness utilizing composites [8,9]. Continuous fiber-reinforced polymer (CFRP) composites are very efficient and rigid in structure and have received significant attention due to their widespread interest. Such materials find extensive applications in aerospace, aviation, and civil engineering. One of the possible constraints of conventional manufacturing methods, such as resin transfer molding, is the high cost of rigid molds, rendering small-scale manufacturing less cost-effective. Emphasizing AM as an efficient and economical solution is intended to develop elaborate designs with a more flexible and cost-efficient method by allowing the creation of mold-free and fiber placement customization [10,11]. Most common and widely used additive manufacturing techniques for polymer composites involve material extrusion (i.e., Fused Filament Fabrication (FFF) and Direct Ink Writing (DIW)), vat photopolymerization (i.e., Digital Light Processing (DLP) and Stereolithography (SLA)), polymer powder bed fusion (Selective Laser Sintering, SLS), and material jetting (droplet-based inkjet printing). They have been instrumental in both the production of complex composite parts, the elimination of the need for molds, waste reduction, cost reduction in labor, and error reduction in production. They enable the capability of accurately controlling filler materials as well as facilitate the creation of polymer composites with functional features of a desired design that would otherwise have been too difficult to accomplish [12,13]. In that regard, AM provides a more flexible and cost-efficient method by allowing the creation of mold-free and fiber placement customization, but difficulties in scaling and consistency are still present [14,15,16,17].

Simultaneously, market trends have increasingly focused on sustainability, where the use of biodegradable polymers, recycled feedstocks, and bio-based reinforcers is being increasingly implemented under the regulation of environmental concerns and lifecycle [18,19]. PLA is heavily utilized in industry among biodegradable polymers, due to excellent life cycle efficiency, decreased logistics demands, and decreased logistics demands. Reinforcing PLA with natural fibers increases its effectiveness for load-bearing applications [20]. Four-dimensional printing is a continuation of 3D printing that introduces the fourth dimension of time so that structures printed can alter their shape or functionality in response to the surrounding environment, e.g., water, light, heat, pH, or magnetic flux. This has been made possible by smart materials that are programmable and will change once fabricated. The latest development with multi-material 3D and 4D printing has greatly expanded the level of customization and performance and has overcome the constraints of traditional manufacturing by blending polymers, metals, ceramics, and biomaterials [21,22]. Layer-by-layer planning and path-control methods with optimization are essential for improving the additive manufacturing process of polymer composites, especially in complex and highly curved shapes that are difficult to produce through the traditional taping, laying, or lamination methods. Process-assisted methods that include ultrasonic embedding incorporated in tailored FFF systems have also been found to enhance interfacial bonding and fiber-matrix bonding to a significantly greater degree, leading to a polymer composite assembly with high mechanical reliability and structural integrity [23,24,25].

**Figure 1:**
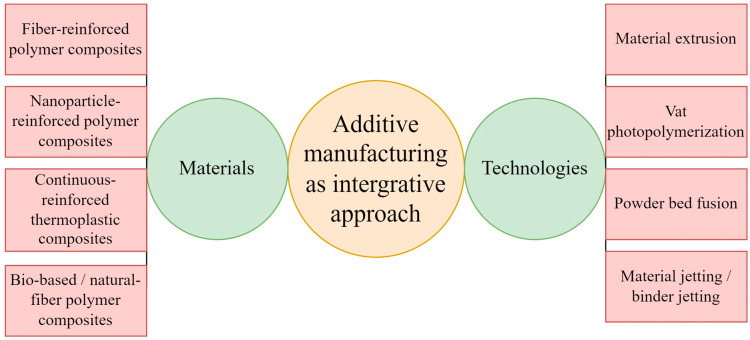
Key materials and processes of polymer composites in additive manufacturing.

Soft robots and actuators can also be developed with the help of multi-material AM. Numerous review articles have investigated 3D printing of fiber- and particle-reinforced polymer composites, as well as the flexural impact behavior of these materials. A broad body of literature also discusses 3D/4D printing of stimuli-responsive polymers, smart magnetic materials, hydrogels, and their application in biomaterials [26]. Laminated composite structures (LCS) comprise successive layers of assorted materials with their structure and make-up adjusted to fit particular performance demands. The material ratios, the layer configurations, and design parameters are key factors that have a significant impact on the overall behavior of these multi-material laminated composites. The current state of high multi-material 3D printing methods provides new opportunities to produce LCS and similar lattice or sandwich structures, commonly known in the literature as panel-based composites [27,28,29,30,31,32]. Over the past years, there has been a high emphasis on the creation of environmentally friendly materials for additive manufacturing, especially the use of biowaste-based fillers in place of traditional polymer filaments. Nonetheless, in spite of their benefits in sustainability, these bio-based materials tend to have processing and printability issues that are comparable to those of recycled plastics, such as inconsistent melt flow, poor interlayer bonding, and variable mechanical performance [33,34]. Figure 1 demonstrates key materials and processes for polymer composites in additive manufacturing as an integrative approach.

Most existing review papers on 3D-printed polymer composites focus on either material type (e.g., fiber-reinforced or stimuli-responsive systems) or additive manufacturing methods. As a result, material architecture, processing strategy, multi-material/graded design, and AI-assisted optimization together are rarely discussed within a single framework. In addition, many reviews do not sufficiently highlight the practical barriers that still limit high-performance and industrial adoption of polymer composite AM. These major challenges include weak interfacial and interlayer bonding, non-uniform filler/fiber dispersion, uncontrolled microstructure evolution during printing (e.g., voids, fiber orientation changes, crystallinity variations), and limited scalability and repeatability when moving from lab-scale demonstrations to reliable large-scale production. Therefore, this review integrates materials, processes, and emerging digital/AI tools while critically discussing these unresolved issues to guide the development of the next generation of high-performance polymer composite AM systems.

## 2. Classification of Polymer Composites in Additive Manufacturing

### 2.1. Carbon Fiber–Reinforced Polymers (CFRP)

#### 2.1.1. Continuously Reinforced Composites

**Figure 2:**
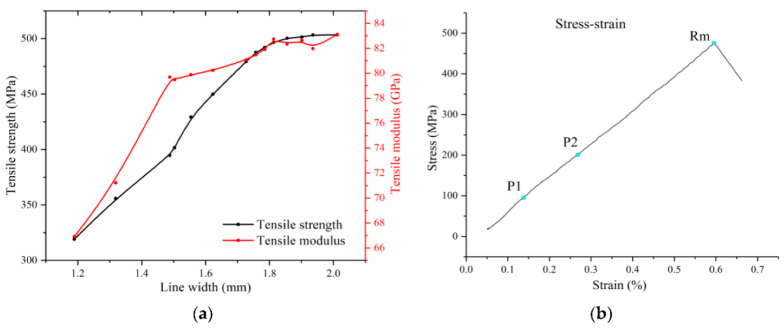
Tensile properties of printed specimens: (**a**) tensile strength and modulus with different line widths; (**b**) stress vs. strain curves of specimens with 1.785 mm line width [49].

AM is transforming the manufacturing sector with the use of thermoplastics and thermoset matrices reinforced with Continuous Carbon Fiber Reinforced–Polymers (CCFRPs). This shift is not only driven by innovation but also by the growing need for sustainable solutions. Efforts to create printers that can process recycled plastics support a circular economic approach. Integrating polymer matrices into 3D-printed CCFRP parts has significantly improved mechanical performance. For example, fracture toughness has reached 2.71 ± 0.25 $MPa\cdot m^{1/2}$ and bending strength 123 ± 15 MPa, highlighting its effectiveness in advancing eco-friendly manufacturing. AM is reshaping conventional methods, including commingled yarns, film stacking, co-woven yarns, and powder impregnation, enabling faster and more flexible design and production [35]. While CCFRPs may be created using diverse manufacturing techniques, AM has unique benefits that can be attributed to its design versatility, the possibility of mass customization, and flexibility. In contrast to conventional methods of manufacturing, AM utilizes various innovative applications, among them the use of continuous carbon fibers via two nozzles (one extruder with thermoplastic, one with fiber) (placed in a polymer matrix), DIW of short carbon fibers, and SLA methods in which short fibers are suspended in resin and cured with UV light. Other AM techniques include laminated object manufacturing (LOM) using pre-impregnated carbon fiber filament sheets, one-screw extrusion of resin-prepreg filaments, and AM systems based on ultrasonic assistance [35,36,37]. CCFRP is extensively used across aerospace, automotive, and petrochemical industries, thanks to its superior strength-to-weight ratio and stiffness compared to traditional metals and alloys. Recently, the use of FFF in the AM of CCFRP has drawn growing interest in both academic and industrial circles. This is largely because FFF does not require molds and offers benefits like reduced production costs, lightweight components, and excellent mechanical strength [38,39,40,41]. The reinforcement is a continuous carbon fiber that is mixed with a plastic matrix to form a CCFRP. The matrix is important as it holds the carbon fibers together so that the structure, ensuring long-term structural stability, and safeguarding the fibrous against the environment such as corrosion, wear, and degradation. Widely used thermoplastic materials in AM as a matrix are nylon (PA), polylactic acid (PLA), acrylonitrile-butadiene-styrene (ABS), and polyether-ether-ketone (PEEK). Such materials are desirable due to their good processing properties and good engineering performances [42,43,44]. So far, the research on the AM of CCFRP has mainly focused on its mechanistic performances, expressed as the improvement of mechanical properties. Various methods have been experimented with in order to achieve this by printing in vacuum environments, applying heat during the printing process, annealing as a post-processing and incorporating compaction during fabrication. These techniques have resulted in apparent enhancements in the strength and general mechanical behaviors in CCFRP components produced via AM [45,46]. Li et al. enhanced the printing speed by applying the tool of microwave to create eddy currents in the carbon fiber which consequently led to the melting of the resin material more efficiently [47]. In a similar fashion, Tu et al. improved the printing process through heating of the carbon fiber using lasers such that the resin would melt faster and printing would be faster in general [48]. The tensile characteristics of CCFRP that is obtained through the multifilament AM should be systematically tested in different line widths. The widths indicate a combination of various key printing parameters and are very important in determining the possibility of high-throughput prototyping in CCFRP. As Figure 2a shows both tensile strength and tensile modulus tend to increase with line width. It is interesting to note that tensile strength is almost proportional to line width at the beginning when it is less than the starting distance between the filaments (1.5 mm). Once the line width approaches 1.5 mm, the tensile strength growth rate starts to pick up a bit, and tensile modulus growth starts to level off. The two properties enter a plateau beyond 1.8 mm, and their rates turn towards zero.

Figure 2a reveals that the specimen whose line width is 1.189 mm seems to have experienced cross-sectional failure following the breaking in the middle when it was subjected to a tensile force of about 9.318 kN. In comparison, Figure 2b indicates that specimens that have wider lines such as that printed at 1.785 mm are more likely to break down in a more brittle mode, perhaps because of more tensile stretching than localized tearing. With the tensile strengths of between 319.06 MPa and 503.4 MPa and the moduli of between 66.9 and 83.11 GPa, the stress-strain curves obtained of this broader specimen at the print temperature of 210 degree Celsius and a rate of 2.5 mm/s and a pressure of 4.5 N (between three filaments) are shown below: The values though relatively high, probably represent a compounding interaction between material consolidation and internal structural defects. Voids, more specifically, appear to be an important factor, particularly when the specimens are narrow, and their concentration would consequently be greater, thereby allowing early shear-type failures to form. This may not come as a shock, as the narrower the line width, the more inter-filament bonding may be hampered, and the fewer avenues may exist to redistribute stress. Nevertheless, the relationship between void formation and mechanical response is likely not linear, and other printing parameters, which were not completely controlled during the current experiment, affect it [49].

#### 2.1.2. Nylon 6,6 with 30 wt% CF

A popular synthetic polymer is nylon, which is particularly famous in the automotive industry as a fiber-based component [50]. The flexibility and durability of nylon, as a product of its synthetic nature, make it suitable for applications across many sectors. It is lightweight, corrosion-resistant, provides good insulation, and does not wear out easily under mechanical and chemical pressure. These characteristics have seen it being used increasingly in modern technologies in the form of circuit breakers, electrical wiring, motors, printed circuit boards, and sensors. Nylon is now even more useful with the emergence of AM, which makes the creation of complex and high-performance parts more design flexible and efficient [50,51,52,53]. However, the use of nylon-based materials is limited, as they lose performance under prolonged exposure to high temperatures. In order to eliminate this shortcoming, scientists have concentrated on enhancing characteristics of nylon, such as thermal conductivity and mechanical strength, using different fillers, such as metal particles, carbon, and ceramics. Such additives are used to make superior-performance reinforced nylon composites. Specifically, nylon 6,6 composites have been greatly enhanced in terms of heat resistance, mechanical strength, and wear durability, which made them applicable in more sophisticated applications [50,51]. Table 1 indicates the essential printing parameters that were applied to produce the Nylon 6,6 and 30% carbon fiber-filled nylon structures. Most settings, such as print speed, infill pattern, and others, were fixed, but some, including temperature and flow rate, were varied, probably to align with the extrusion characteristics of materials.

### 2.2. Glass Fiber–Reinforced Polymers

Scientists have explored the glass fiber–reinforced polypropylene (GFRPP) composite made in the laboratory, addressing the impact of fiber length (0.1–50 mm) and concentration (3–60% by weight) on the composites. Their results indicate that stiffness, dimensional stability, and warping resistance are greatly enhanced by increasing the fiber content. When the fiber content increases to 40%, the heat deflection temperature also increases to approximately 149 °C (300 °F), and the thermal expansion rate is reduced two-fold. Also, the tensile and flexural strength are significantly higher when chemical coupling agents are utilized in contrast with conventional GFRPP [54]. The applications of GFRPs are prevalent in the aerospace and automotive industries because of their strong and lightweight characteristics. The increasing demand for customized composite structures has, however, brought out the weaknesses of traditional molding processes, which are costly and time-consuming to develop. This has given birth to the emergence of AM of fiber-reinforced polymer composites (FRPCs), which are lighter, less expensive, and have higher levels of automation. In contrast to traditional techniques, AM needs minimum post-processing and enables novel reinforcement approaches like the incorporation of circumferential fiber rings and clustered fiber layers to strengthen and improve the functionality of the end components [53,54,55,56].

### 2.3. Aramid Fiber Composites

The fibers used in AM composites are the same as those used in composite manufacturing of carbon fiber, glass fiber, aramid fiber, and natural fibers such as flax. Aramid fibers are not widely used in traditional composites nowadays, but they are especially effective with AM [57,58]. They have great bending strength and a high abrasion level, which lowers the chances of fibers being broken during printing, particularly when going around sharp corners or narrow bends. This renders the aramid fibers more beneficial in AM, where these complicated geometries are more prevalent than in established procedures [57,58,59,60].

**Figure 3:**
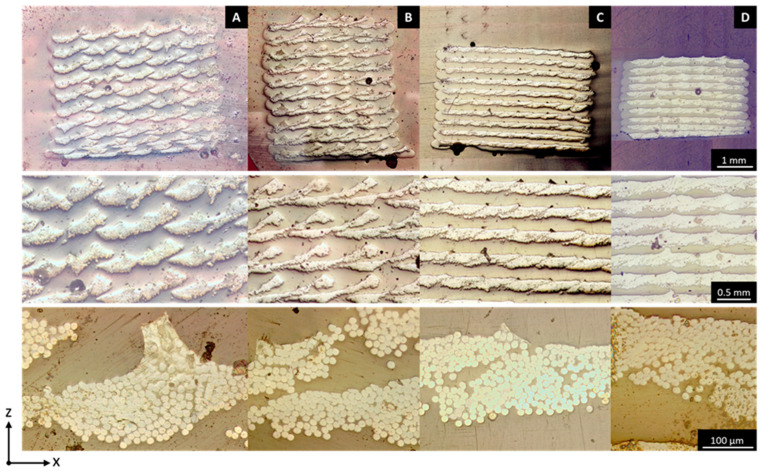
Microscopic analysis of cross-sectional cuts of aramid composites; (**A**) 20 vol%, (**B**,**C**) 25 vol%, and (**D**) 45 vol%. An evolution towards a continuous fiber-reinforced layer can be seen when going towards higher fiber content, where printed lines overlap, and the layer height is reduced [61].

In AM, the layer height and the width of a line can be adjusted in order to print various microstructures and modulate the fiber content of the printed composite. Figure 3 shows the various microstructures as the printing setups vary in four different setups:

- Configuration a: Using a large layer height and wide line spacing creates a distinct array of individual fiber bundles, each surrounded by the polymer matrix.
- From a to b: Reducing the line spacing causes the lines to start overlapping within the same layer. However, since the layer height remains large, the new lines do not fully merge with adjacent ones; instead, they are layered on top, with gaps in between filled by the matrix.
- From a to c: Lowering the layer height increases compaction, pressing the lines more firmly into one another. This results in a more organized, layered structure, closely resembling traditional laminated composites.
- From c to d: Further reducing both the layer height and line spacing enhances compaction even more. This minimizes the resin-rich layers between the layers from about 138 ± 13 µm to 88 ± 24 µm, boosting the fiber volume fraction and eliminating large interlayer voids.

### 2.4. Natural Fiber–Reinforced Composites

Natural fiber composite filaments with their strong mechanical characteristics, environmental friendliness, and renewable sources have received significant attention over the last few years [62]. The most interesting development in the sector is the incorporation of natural fiber composite filamentation in AM. This new paradigm combines the environmental and sustainability of natural fibers with the ability to make designs and the precision of the AM process [63]. Initially, FFF was applied to make low-stress prototypes, like domestic objects and toys, and its usage has since expanded radically. Another field of improvement is the creation of natural fiber composite filaments based on materials such as hemp, flax, or bamboo. The filaments have better mechanical properties, create less pollution, and have a broader application. With the rise in the trend of manufacturing eco-friendly products, natural fiber composites are fast becoming a formidable substitute for traditional filaments made of polymer. Their application in the field of AM, blending the sustainability of renewable fibers with the flexibility and accuracy of current AM methods, is one of the most promising developments [64,65,66,67,68]. Natural fibers are naturally hydrophilic, i.e., they absorb water, potentially causing the degradation of composite materials with time. Researchers are considering using hydrophobic coatings and incorporating moisture-absorbing substances (desiccants) in the material content to solve this problem. In spite of this, natural fibers are still a cost-effective material that is commonly used in various industries, such as packaging, automotive, construction, and even in interior parts of railway coaches and warehouses. They are also increasingly being used as a cheaper substitute for glass fiber. Nevertheless, the disadvantage of the NFRCs is their low mechanical strength. In order to solve this, hybridization, which involves the integration of various kinds of fibers, is being resorted to in order to improve their mechanical performance and increase their potential applications. Moisture absorption behavior is also subject to this process. As a case study, the absorption rates of water in bidirectionally woven and hand-layup compressed composites were 2.8% in hemp/flax/epoxy, 3% in hemp/jute/flax/epoxy, and 4.5% in hemp/jute/epoxy blends [69,70]. This has been found to be caused by the fact that flax fiber composite absorbs significantly more moisture, up to 12 times than glass fiber composite, when resin is infused under vacuum using vacuum-assisted resin infusion. This is also considered one of the largest limitations of natural fibers because of the high amount of water absorption. To reduce this issue, it is necessary that chemical treatment is used to reduce excessive uptake of water. Mechanical performance of the treated natural fibers is greatly affected by the length of the fibers, aspect ratio, quality of bonding between the resulting fiber and the matrix, and many other factors. Some of the common measures that have been applied to improve adhesion of fibers and matrices and reduce innate hydrophilicity of NFRCs that ultimately result in increased durability and strengths include alkaline, silane, acetylation, benzoylation, and peroxide modification [71,72]. It should also be mentioned that the discrepancy of mechanical characteristics is quite a common phenomenon in natural fiber composites. These variations can be found in different aspects, which may include processing conditions modification, manufacturing, fiber treatment, and different sources of fibers and polymer matrices. The hemp fiber studies that have been reinforced by poly-lactic acid (PLA) matrix have been very informative. The addition of hemp fibers tended to increase the elastic modulus regardless of the fiber content and pretreatment modes. Interestingly, the tensile strength and tensile modulus tended to reduce with the rise in fiber content. Even though pretreatment and fiber loading levels did not play an important role in flexural modulus, alkali and silane treatment with hemp fiber at 30 percent and 50 percent had a greater impact on the flexural strength of composite materials [73]. Table 2 enlists recent works related to the optimization of natural fibers with matrix material.

### Limitations of Natural Fiber–Reinforced Polymer Composites

Although natural fibers (e.g., jute, flax, hemp, kenaf, sisal, bamboo) offer clear benefits in terms of sustainability, low density, and reduced environmental burden, they exhibit inherent limitations that must be critically considered for realistic engineering deployment in AM polymer composites. Compared with synthetic fibers (carbon or glass), natural fibers generally provide lower tensile strength, lower stiffness, and reduced fatigue resistance, which restricts their use in high-load structural applications where stiffness retention and long-term reliability are mandatory [69,77].

A major limitation is moisture absorption, arising from the hydrophilic nature of lignocellulosic fibers. Water uptake can cause fiber swelling, microcracking at the fiber–matrix interface, plasticization of the polymer matrix, and gradual loss of interfacial shear strength. As a result, mechanical properties (strength, modulus, impact resistance) may deteriorate significantly under humid or wet service conditions, particularly when printed parts contain porosity or imperfect fusion typical of extrusion-based AM [78,79,80].

Natural fibers are also thermally sensitive. Many lignocellulosic fibers undergo thermal degradation, hemicellulose breakdown, or discoloration at elevated processing temperatures, limiting their compatibility with high-temperature polymers and narrowing the AM processing window. Thermal cycling can further aggravate debonding due to differential thermal expansion between fiber and matrix, especially when combined with moisture-driven swelling/shrinkage.

Another critical issue is variability in properties. Fiber quality depends on plant species, harvesting season, retting/extraction route, fiber diameter distribution, and storage history. This natural variability makes it difficult to ensure consistent filament quality and repeatable printed part performance across batches, complicating certification pathways for engineering components.

### 2.5. Polymer Nanocomposites

#### 2.5.1. Carbon Nanotube (CNT) Composites

Carbon Nanotubes (CNTs) refer to an extraordinary filler employed in polymer-based composites. CNTs are considered to be one of the best reinforcement materials, thanks to their extremely high aspect ratio and amazing mechanical properties, such as tensile strength that ranges between 10 and 63 GPa and a Young’s modulus of between 0.3 and 1 TPa. They also offer impressive physical properties, including electrical conductivity in the range of $10^{6}\;to\;10^{7}$ S/m. Due to their peculiar structure and properties, CNTs are able to add new functions to epoxy composites, such as vibration damping, electrochemical sensing, and electromagnetic interference (EMI) suppression. In comparison to other carbon-based nanomaterials. such as graphite and graphene, CNTs have a far lower percolation threshold (approximately 0.1 wt%); therefore, an insignificant amount of material is required to create a connective network. Their one-dimensional (1D) structure has also provided additional mechanical advantage, which contributes to their special utility in composite applications [89,91].

#### 2.5.2. Graphene-Based Composites

**Figure 4:**
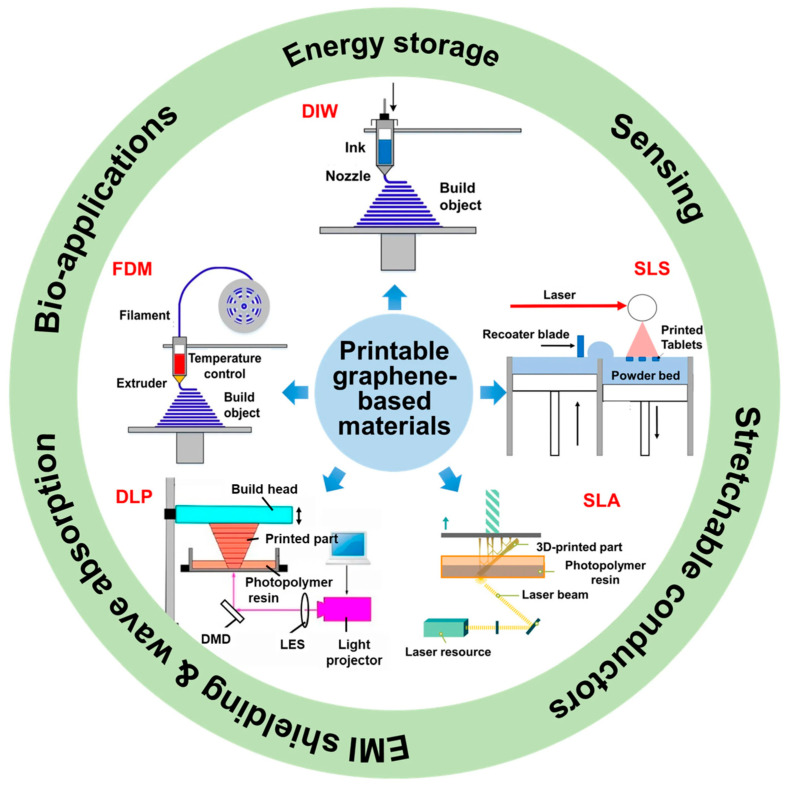
Overview presentation of printable graphene-based materials [96].

During the last decade, the field of 3D printing of structures based on graphene materials has actively developed, with many more intricate and high-performance structures being developed. Strategies such as extrusion-based, photopolymerization, and powder-based 3D printing have turned out to be useful in the production of pure graphene and graphene/polymer composite materials [92,93,94,95]. Our paper demonstrates the formation of complex structures of graphene, including functionalized composites, porous scaffolds, and lattices with controlled porosity, developed through a number of approaches based on 3D printing. With such capabilities, these architectures are of great potential in a wide range of applications, including storage of energy, sensing, conductivity, wave absorption, and biomedical applications, all of which have been made possible with the help of carefully designed materials and processes. Figure 4 demonstrates a compilation of the important printing methods and multifunction of graphene and graphene/polymer composites.

DIW and FFF are 3D printing methods based on extrusion, in which layers of material are deposited in the shape of successive layers by forcing a material in a semi-liquid or molten form through a nozzle. Such processes appear to be rather versatile, as they can take a very broad selection of feedstock: thermoplastics, thermosets, bioinks, pastes, and even low-melting-point metals can be used. With that said, they do not lack trade-offs. They have some limitations in resolution, and the end structures might not have the finer architectural details that can be produced using other methods. Conversely, situations that involve photopolymerization, like SLA and DLP, are based on UV light to cure liquid resin selectively. These systems are commonly touted to be fast and accurate, particularly in making complex geometries. But again, it is not all upside. The selection of compatible materials is also fairly limited, the size of prints is often limited, and equipment is not quite cost-efficient. Another pathway is powder-based methods where thermoplastic polymer-based graphene composites are used, e.g., powder bed fusion. Although they appear good with more advanced material systems, scaling, cost, and consistency of result properties remain an issue. It is interesting to note that each of these approaches can be placed into the wider discussions of performance trade-offs between AM resolution and scalability, and versatility and specialization [95,96,97,98,99,100].

### 2.6. Elastomers and Rubber-like Polymers

Over the past years, elastomeric syntheses have already shown great potential in wearable electronics, soft robotics, and real-time health monitoring, where flexibility and responsiveness are highly imperative. Their growing application in medical sensing and AI-assisted devices is indicative of a larger overall pattern of using materials that can mimic biological softness and adjust to mechanical stress [101,102,103,104]. Rubber, especially elastomers, might have to possess specific customized properties depending on the context. The use of magnetic or electrically conductive fillers has proven to be one effective approach for tuning a material’s responsiveness to external fields; however, this activity frequently depends on the dispersal uniformity of the fillers and the interactions the fillers have with the underlying matrix [105,106]. When certain fillers are used together with some rubber composites, they appear to have a great potential for absorbing electromagnetic waves, and in fact, they may be used as transducers, where movement can be converted into tiny bursts of electricity, which can be useful in low-power systems, but the practice of this still has several challenges. It is proposed by Fasolt et al. [107] that breakdown voltage in dielectric elastomer actuators can be affected by the use of electrodes. They report that this voltage threshold could be reduced by stiffer, silicone rubber-based electrodes, but how well this is generalized to other actuator designs is not quite clear. Apparently, harder electrodes are more beneficial, but other considerations, such as electrode sticking and flexibility, might also be contributory, according to some researchers in related discussions. Wang et al. [108] investigated blends of methyl vinyl silicone rubber and fluorosilicone rubber, and their findings appear to indicate that it enhances the mechanical power, dielectric properties, and surface hydrophobicity. With that said, the level of improvement is probably dependent on mixing ratio and processing conditions that are not always comparable across research. This approach is still quite promising, but may still have questions of cost or compatibility, or long-term stability, which still appear in larger material research. Razzaq et al. [109] examined 4D printing electro-active composite based on polyester urethane (PEU), polylactic acid (PLA), and multiwall carbon nanotubes (MWCNTs). These are the materials that can be used in FFF and appear to exhibit a triple-shape memory effect, in response to resistive heating. Although the work indicates potential uses in systems such as space systems or soft robotics, the degree to which this effect may be reliable in the conditions operational in the real world is a little unclear. Kumar et al. [110] conducted tests with silicone rubber composites, the addition of graphene nanoplatelets (GNPs), and electrolyte-iron particles (EIPs) with the aim of producing materials capable of stretching and following magnetic fields. This was cured at room temperature, which makes things practical, but what impressed me was how GNPs appeared to increase the stiffness of the material without rendering it brittle. It is suggested that this combination could be used to trade off between mechanical strength and flexibility, which is often difficult with the elastomers. With the introduction of EIPs, the composites were seen to experience a clear increase in magnetic responsiveness as well, perhaps applicable to soft robotics. With that said, these benefits may be rather dependent on the processing specifics or material proportions, but these were not dug into. Though the results are encouraging, they likely leave a few questions unanswered in terms of long-term performance and repeatability in a real-world setting. Even though 3D printing of thermoplastic elastomers and some low viscosity reactive elastomers has been more or less successful, AM reports on thermoset elastomers have been mostly limited to fully compounded reinforced thermoset systems, and AM reports on conventional rubber compounds remain scarce. These are materials comprising the base elastomer, fillers, and a curing system, usually specific to the end-use application. Their viscous properties, contraction nature, and thermosetting properties render them especially challenging to handle using AM methods. Nevertheless, an increasing industrial interest is taking these challenges. This space has begun to be ventured into with some success reports of printing materials such as nitrile butadiene rubber (NBR), ethylene propylene diene monomer (EPDM), and fluoroelastomer blends. Even though the initial outcomes appear encouraging, the technical obstacles indicate that wider usage is perhaps still ahead [107,108,109]. It was reported by Leinweber et al. [109] that a 3D printing system using a corotating twin-screw extruder and an FFF head can enable the printing of carbon black-filled rubber and thermoplastics to be possible. Although the configuration probably aids in processing more sophisticated or filled materials, even the degree of reliability of the setup in the face of different formulations remains unclear. Rubber 3D printing is yet to reach that point, but there are some broad understandings that are already starting to form the picture of what an ideal process should look like. A 3D printing apparatus with a co-rotating twin-screw extruder and an FFF print head can be used to produce carbon black-filled rubber as well as thermoplastic composite materials. Even though such a structure allows working with complex or densely filled formulations, the overall stability of the structure concerning various material compositions is an open question.

**Figure 5:**
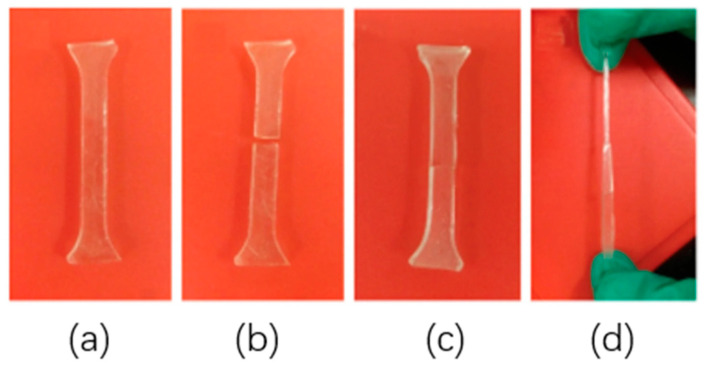
Representative photographs of an elastomer specimen after processing and curing: (**a**) intact sample, (**b**) sample after cutting, (**c**) sample after self-repair following curing at 80 °C for 24 h, and (**d**) specimen under continuous tensile loading, showing necking without fracture [110].

Figure 5 presents a representative experimental demonstration of elastomer processing and post-curing behavior. The printed elastomer specimen was cured in an oven at 80 °C for 24 h and subsequently subjected to tensile loading. The results show that even after significant necking, the specimen did not fracture, indicating that the curing process preserved the mechanical integrity of the material [110].

Since viscosity plays a major role in how well these materials can be processed and extruded through a nozzle, selections were made to span a range of Mooney viscosities. NBR, with noticeably lower viscosity than NR, and EPDM, with much higher viscosity, were included to reflect this variation. Table 3 summarizes the Mooney viscosity values and vulcanization conditions provided by the manufacturer [111]. All materials were supplied as rubber sheets and then cut into strips of approximately 50 × 5 × 2 mm^3^ for use in the test setup.

### 2.7. Metal Fiber–Reinforced Polymer Composites

Metal fiber–reinforced polymer composites are polymers that are reinforced with short metal fibers (e.g., steel, copper, aluminum), resulting in anisotropic strength, increased thermal conductivity, and better performance, without compromising polymer AM processability [112]. The use of metal fibers allows one to achieve a set of properties that is unique and bridges the gap between all-polymeric composites and metal-polymer hybrids. Metal fibers have a high aspect ratio and, therefore, can be developed as continuous or semi-continuous conductive and load-transfer networks at relatively small volume fractions, which results in enhanced stiffness, wear resistance, and heat dissipation. These features make the metal fiber–reinforced composites unique to the carbon- or glass-fiber systems when thermal management, electrical functions, or reinforcement in a specific area are needed [113,114]. The extrusion-based metal fiber–reinforced polymer composite additive manufacturing techniques that have been mostly demonstrated are FFF and DIW technologies [115,116]. Metal fibers in these processes are short or micro-scale and are homogenously distributed throughout thermoplastic or viscoelastic polymer matrices, which are deposited layer-by-layer. The main problems are poor dispersibility of fibers, overwear of the nozzle, augmented viscosity of the melt, and a lack of interfacial strength between the metal fibers and the polymer matrix. With the proper surface treatments to the metal fibers, along with process parameters, fiber dispersion, interfacial bonding, and mechanical reliability of the resultant composites can be greatly improved [117,118,119]. Recent research proves that metal micro-fiber reinforcement is effective in increasing thermal conductivity and stiffness while still retaining sufficient toughness and printability. Copper- and stainless-steel–reinforced polymer composites have high promise of application in heat dissipation assemblies, in interference-shielding of new electromagnetic fields, and in structural parts whose mechanical and thermal performance are needed at the same time. Nevertheless, in comparison to carbon-based reinforcements, the role of metal fibers increases density and galvanic corrosion potential risks in the aggressive service environments, which is why these factors are paramount to consider during material selection [120,121,122].

### 2.8. Polymer Blends and Alloys

**Figure 6:**
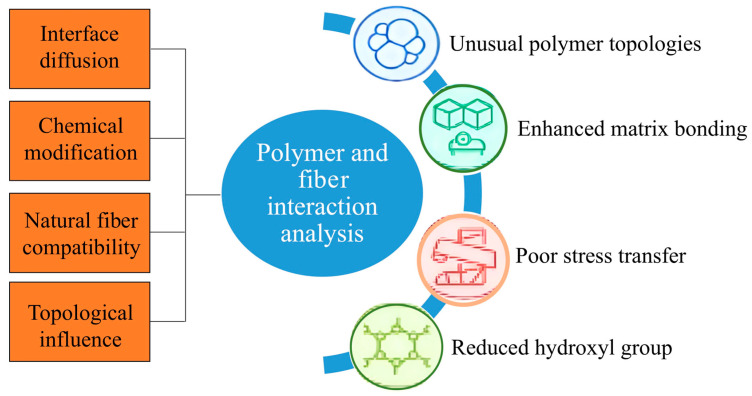
Polymer and fiber interaction analysis highlighting four interaction domains.

The idea of tuning surface properties by using polymers with unusual topologies has long drawn attention, though the exact mechanisms driving surface segregation are still not fully clear. Part of this curiosity likely arises from how topology appears to influence both the structure and dynamics of polymer systems, sometimes in ways that are not easily predictable and may depend on subtle molecular interactions [123]. Research may suggest how the repetitive chemical units in polymer chains affect interface diffusion; however, further studies are still needed on molecular weight and architecture in relation to preferential adsorption [124]. For linear and cyclic polymer blends, self-consistent field theory may suggest that cyclic polymers will aggregate more at the interfaces, regardless of their molecular weight, although experimental observations have disputed this theoretical expectation [125]. The combination of natural fibers and polymers raises a lot of concern, especially due to their varying chemical structures that are quite different. This dislocation may disrupt the transfer of stress at the interface. To solve this, fiber treatment with a reactive functional group is usually investigated. These adjustments can assist in minimizing moisture absorption and enhancing, at least partially, the compatibility between fiber and matrix, but it does not appear that adhesion perfection can be reached [126]. According to Kabir et al. [122], chemical treatment is important in the processing of natural fibers. It seems that these treatments can decrease hydroxyl groups, and this causes a decrease in fiber hydrophilicity, potentially resulting in an increase in mechanical strength and dimensional stability. However, the magnitude of such improvements will be dependent on the applied methodology. Figure 6 outlines polymer and fiber interaction analysis, which highlights four interaction domains such as interface diffusion, chemical modification, natural fiber compatibility, and topological influence.

## 3. Functionalized and Advanced Behavioral Polymer Composites

Specialized polymer composite materials in additive manufacturing are designed to produce functional performance in application-specific ways, in addition to standard structural functions, such as controlled degradation, biological interaction, and stimuli-responsive behavior. According to the functional intent, such materials are typically subdivided into biodegradable composites, biomaterials, biocompatible systems, and intelligent or stimuli-responsive polymer composites [33]. Even though most polymers and composites can be bio-compatible or recyclable, engineered functionality, which can be controlled by biodegradation, biological interaction, or stimuli-responsive behavior, defines specialization. According to functional intent and application relevance, specialized polymer composite materials in AM can be subdivided into biodegradable composites, biomaterials, biocompatible composites, and intelligent or stimuli-responsive composites [127,128,129,130].

### 3.1. Biodegradable Polymer Composites

Biodegradable polymer composites are materials that can be degraded under biological, chemical, or environmental conditions after their service life. These materials are commonly derived from aliphatic polyesters, such as polylactic acid (PLA), polycaprolactone (PCL), and polyhydroxyalkanoates (PHA), and commonly filled with natural fibers or bio-derived fillers [131]. Mohiuddin et al. [132] established that biodegradable graphene nanocomposites have a combination of multifunctional attributes that include increasing the mechanical strength, bioactivity, and controlled degradation. These properties allow them to be useful templates for additively manufactured biomedical scaffolds and drug delivery systems. The main features of biodegradable composites are low density, intermediate mechanical strength, adjustable degradation rates, and the ability to be used in extrusion-based AM. They should be used in short-life products and applications that are environmentally sustainable due to their controlled degradation behavior. Examples include PLA–cellulose fiber composites for packaging and disposable components, and PCL-based composites for temporary biomedical scaffolds [133,134].

### 3.2. Biomaterials and Biocompatible Polymer Composites

Biomaterials are polymer composite systems particularly designed to react in a safe and effective way in biological environments. Most polymers can be biocompatible, whereas biomaterials designed to act biologically are characterized by biological functionality, e.g., tissue integration, bioactivity, or controllable biological response [135,136]. These composites focus on cytocompatibility, controlled surface chemistry, and are, in certain instances, bioresorbable. Additive manufacturing allows the geometrical, pore, and internal architecture to be controlled precisely, which is paramount to biomedical performance. Applications: PLA-hydroxyapatite bone tissue engineering composites and polyethylene glycol (PEG)-based soft tissue applications and medical devices, photopolymer composite [137,138].

### 3.3. Intelligent and Surveillance Polymer Composites

Smart polymer composites are the materials whose behavior with regard to temperature, electrical fields, magnetic fields, moisture, or mechanical stress can be predicted and reversed. Such responses enable sensing, actuation, or adaptive behavior at the component level [139]. Other useful fillers that are often used in such composites include carbon nanotubes, graphene, or shape-memory additives. AM enables the localized control of material composition and architecture, enabling localized functionality and multifunctional integration of a single printed structure. Applications include adaptive component shape-memory polymer composites, strain-sensing structures, and electrically responsive CNT-reinforced polymers [140,141].

### 3.4. Smart and Stimuli-Responsive Composites

#### 3.4.1. Shape-Memory Polymers

Shape memory polymer composites (SMPs) have a shape memory effect, meaning they can return to their original shape after being heavily deformed when triggered by a certain stimulus. The mechanical and thermal characteristics of SMPs can be notably enhanced by external reinforcements. Boudjellal et al. [142] explored the reinforcement of SMPs through a range of fibers and compounds, as well as stimulation methods such as heating, lighting, and the use of solvents. Different activation techniques and mechanisms for SMPs are illustrated in Table 4.

**Figure 7:**
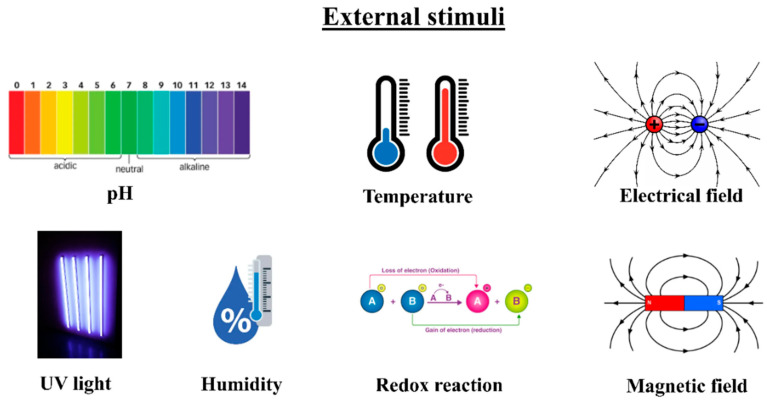
External factors for activation of polymer actuators [146].

Multi-functional polyurethane (PU) composites were enriched by graphene oxide (GO), which possesses integrated self-healing and shape memory properties, alongside improved mechanical performances [144]. Dayyoub et al. [143] examined the essential aspects of three basic types of external inducers (thermal, chemical, and light), emphasizing what drives SMPCs’ performance, along with potential application areas. Thermo-responsive SMPs activate their shape memory behavior using various heating methods like direct, indirect, inductive, and electro-resistive heating, depending on how they are being used. Photo-responsive and photo-thermal SMPs work through light-sensitivity, but their activation systems are more complex and have stricter processing requirements. In chemo-responsive SMPs, solvent molecules become absorbed into the polymer matrix, which breaks the secondary bonds, boosts chain mobility, and shortens the relaxation time, causing the polymer to swell and return to its original shape [143,145]. Figure 7 displays various external inducers that lead to the activation of polymer actuators.

#### 3.4.2. Piezoelectric Polymer Composites

**Figure 8:**
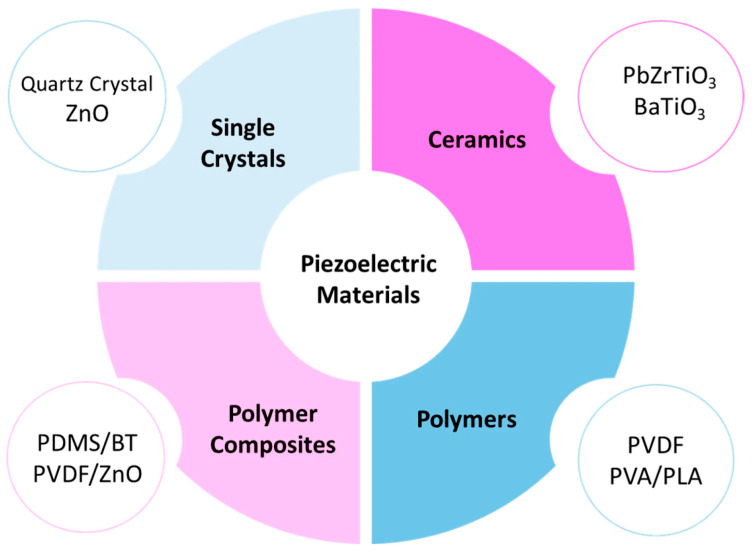
Various classes of piezoelectric materials [151].

The research by Chang et al. [147] is focused on creating 3D-printable ceramic–polymer composites with high printability and strong piezoelectric responses for flexible tactile sensors and self-powered electronics. The primary piezoelectric material used is Pb[(Mg_1/3_Nb_2/3_)_0.1_Zr_0.45_Ti_0.45_]O_3_ (PMN–PZT) ceramic powder. To refine its traits for 3D printing and piezoelectric response, PMN–PZT powder was treated with 3-(trimethoxysilyl) propyl methacrylate. Integrating BaTiO_3_ particles into polymer materials creates composites that are both flexible and piezoelectrically stronger than pure polymers, solving the problem of ceramics being too stiff and brittle [148]. Flexible nanogenerators made by Bouhamed et al. [149], combining BaTiO_3_ and polydimethylsiloxane, show that higher BaTiO_3_ content leads to increased voltage and power output. These nanogenerators are highly efficient, converting nearly 80 percent of mechanical energy into electrical energy. They are also lightweight, flexible, and environmentally friendly since they do not need fuel or produce any pollution. Huang et al. [150] introduced a new way of fabricating self-poled piezoelectric polymer composites using melt-state dynamic pressure, which cuts out the need for the conventional, energy-intensive electrical-poling process. The researchers placed a melt-state dynamic pressure procedure on the table, referred to as “Energy Implantation”, to produce self-poled piezoelectric materials. This approach entails the introduction of dynamic pressure during the fabrication process to stimulate self-poling. The different classes of piezoelectric materials are summarized in Figure 8, showing their respective compositions and applications.

#### 3.4.3. Self-Healing Polymers

**Figure 9:**
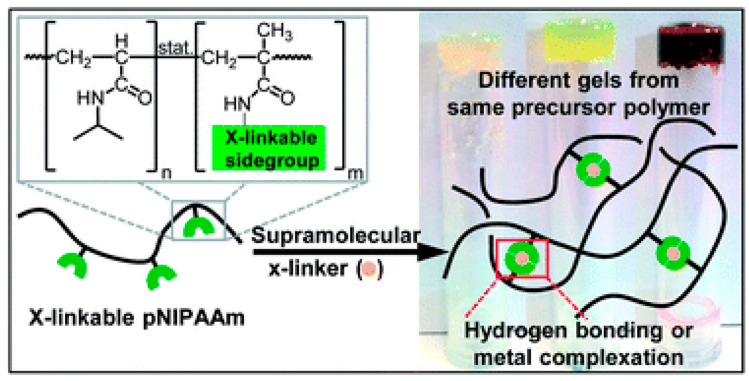
Illustration of non-covalent bonds forming in supramolecular systems [155].

Self-healing composites are intelligent materials that can autonomously restore themselves. They are tailored to address issues like cracks that develop deep inside, which are hard to spot and fix. Graphene’s impressive electrical, thermal, and mechanical properties establish it as a preferred additive for producing self-healing composites that can be employed across multiple applications [152]. Metallopolymers are macromolecules embedded with transition metals that bestow a range of functional traits, including self-healing. A salient type of self-healing polymers, metallosupramolecular polymers, forms through the connection between meta–ligand (M-L) complexation. This method is effective because of the reversibility, high stability, and fast formation rate of coordination complexes [153,154]. Figure 9 provides a graphical representation of the formation of non-covalent bonds in supramolecular systems.

**Figure 10:**
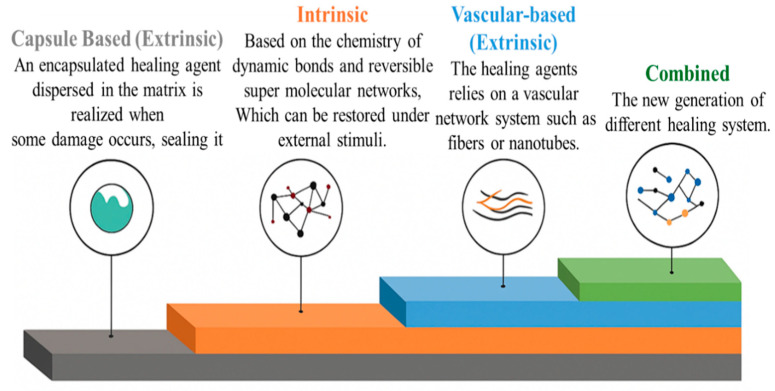
Self-healing methods [157].

The development of self-healing polymer materials primarily depends on two basic strategies—intrinsic and extrinsic self-healing. Intrinsic self-healing operates through the polymer’s internal chemical bonds when exposed to external influences such as thermal energy, chemicals, and ultraviolet lighting. Extrinsic self-healing involves introducing a healing agent into cracks from external containers embedded within the material. These containers burst under the action of a propagating crack, releasing the healing agent to repair the damage [153,156]. An overview of the primary self-healing mechanisms and their operating principles is shown in Figure 10.

### 3.5. Electrically and Thermally Conductive Composites

The brisk progress and pervasive application of electronic gadgets and wireless networks have caused an increase in electromagnetic interference (EMI), a type of electromagnetic pollution that disrupts the normal functioning of electronic devices and can weaken the strength of wireless communication signals. Therefore, developing materials capable of blocking EMI has become imperative to deal with this matter. Polymer-based composites incorporated with adequate fillers have emerged as a major solution to protect against EMI [158,159]. The overall shielding efficiency of polymer composites is driven by a variety of factors, such as permeability, permittivity, filler content, aspect-ratio, size, shape, conductivity, and thickness [159]. Research conducted by Al-Saleh et al. [160] focused on polylactic acid composites that were infused with CNTs, graphene nanoplatelets (GNP), and an equal blend of CNT and GNP. Composites incorporated with CNTs outperformed both GNP-based and CNT:GNP hybrid composites in terms of EMI shielding. Pornea et al. [161] investigated poly(dimethylsiloxane)-based composite augmented with a three-part filler system comprising aluminum oxide (Al_2_O_3_), hexagonal boron nitride, and boron nitride nanotubes. In the hybrid system, aluminum oxide (Al_2_O_3_) was the main filler and functioned like a central node, boron nitride was integrated to generate interconnected heat conduction pathways, and boron nitride nanotubes were incorporated to facilitate phonon transport through diverse directional pathways. Carbon nanofibers have a cone-shaped structure and angled graphite planes, which offer a high surface-volume ratio and effective conductive channels. It has been demonstrated in the past that by adding a low percentage of carbon nanofibers, polymer matrix electrical conductivity can be greatly increased, up to the range of on the order of 10^−1^ to 10^2^ S/m, depending on the quality of filler dispersion [162]. These levels of conductivity are high enough to allow a successful electromagnetic interference shielding due to a combined reflection-dominated and absorption-dominated process [163]. Tran et al. [164] focused on DIW AM of (CNT) composites to generate materials exhibiting enhanced thermal dissipation and EMI shielding capabilities. With 10 wt% loading of CNTs, thermal conductivity improved by 92% over neat phenolic, hitting 0.408 W/m·K. For 5 mm-thick samples with 10 wt% CNT loading, an impressive EMI shielding of 41.6 dB was achieved, offering a shielding efficiency of 99.99%. Carbon nanofibers possess a cone-like structure and angled graphite layers, which provide distinctive mechanical properties and a high surface-to-volume ratio. The report shows that the electrical conductivity of polymers may be significantly enhanced by mixing them with a small quantity of carbon nanofibers [162].

### 3.6. Dielectric and Electronic Functional Composites

HfO_2_ nanoparticles possess a medium dielectric constant and a wide bandgap. Using this as filler with polymer nanocomposites results in both an enhanced dielectric constant and reduced leakage current. Consequently, significant enhancements in electric displacement, discharged energy density, and charge–discharge efficiency at high temperature are achieved by the composites [165]. Nanomaterials exhibit excellent electrical properties because of their distinct behavior at the nanoscale level. Composites incorporating nanoparticles, nanotubes, and nanowires exhibit better control over electrical properties such as conductivity, resistivity, and dielectricity [166].

Geopolymers are known for their strong thermal resistance and durability, but their brittle nature and weak performance under tensile and bending stresses make them unsuitable for certain structural uses where flexibility and toughness are essential. To address this inherent brittleness of geopolymers, considerable research has been devoted to enhancing them with both synthetic and natural fibers. This approach aims to enhance their ductility and tensile strength, making them more suitable for structural applications [167,168,169,170]. Table 5 provides an overview of the notable components of geopolymers.

To improve the structural performance of timber beams, especially under bending forces, pultruded Glass Fiber–Reinforced Polymer (GFRP) profiles are used as reinforcements. These profiles help increase the beam’s load-bearing capacity and flexural stiffness, making them more suitable for demanding structural applications. The effectiveness of the beam depends on the quantity and alignment of the applied GFRP profiles. A hybrid mixture of aramid and basalt fibers can significantly boost the strength and fire resistance of reinforced concrete (RC) elements. Basalt fiber is favored because of its capability to withstand high temperatures and corrosion, while aramid fibers like Kevlar are prized for their exceptional tensile strength, light weight, and outstanding flame-retardant properties [171].

### 3.7. Multi-Functional and Hybrid Composites

Polymer composites may exhibit enhanced properties with the incorporation of high-performance fibers like aramid fiber. These fibers are prevalently used for composite reinforcements because of their excellent comprehensive properties, including low density, high strength, high specific modulus, and high temperature resistance [172]. Metal–Organic Flame Retardants (MOFRs) are a hybrid of metallic compounds and organic components, which perform as a fire safety-enhancing additive of polymer composites. Released toxic gases and smoke during the combustion of polymer composites are suppressed by the metallic part in MOFRs, and the organic part is essential to make links between the polymer matrix and MOFRs through chemical reactions and interfacial interactions, thereby ensuring improved compatibility between both phases [173]. The incorporation of fly ash as filler in pineapple leaf fiber (PALF)–reinforced epoxy polymer composites results in enhanced mechanical strength as well as effective water resistance of the composites. The composite with 20 wt% PALF and 6 wt% fly ash yields the highest tensile strength (86.6 MPa), indicating a 65.3% improvement in comparison to the neat composites [174]. Discontinuous fiber–reinforced polymer composites (DFRPCs) fabricated by material extrusion AM (MEAM) processes like FFF and Large Area AM (LAAM), are widely applied in different fields because of their elevated mechanical characteristics, robust performances, light weight, and ability to form complex structures. DFRPCs produced by the MEAM process are largely employed as finished engineering parts in the aerospace industry. For the rapid fabrication in the automobile sectors, shipbuilding industries, and emergency medical purposes, LAAM technology is specifically useful. LAAM was widely used to rapidly build essential infrastructure for emergency medical purposes during the COVID-19 pandemic [175].

## 4. Additive Manufacturing Techniques for Polymer Composites

Polymer composite AM technologies vary in a fundamental way in the feedstock material form, consolidation mechanism, and reinforcement control, which directly relate to anisotropy, microstructure development, and functional performance. In accordance with such governing mechanisms, the processes belong to four groups: (i) Extrusion-based deposition, (ii) Powder-bed fusion, (iii) Vat photopolymerization, and (iv) Droplet-based jetting. This classification offers an equivalent model of comparing composite printability and performance.

### 4.1. Extrusion-Based Deposition

#### 4.1.1. Fused Filament Fabrication (FFF)

**Figure 11:**
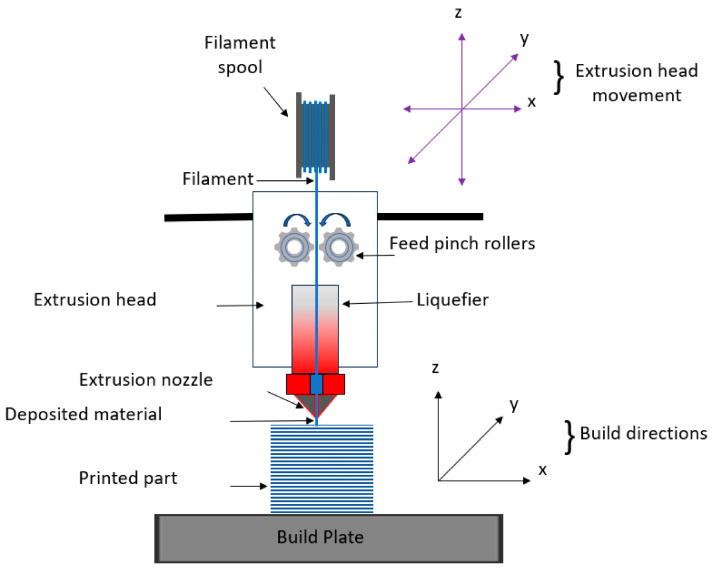
Schematic of Fused Filament Fabrication (FFF) extrusion procedure [179].

FFF is a deposition method that consists of adding the molten thermoplastic filament by means of a heated nozzle to add layers one at a time to create parts [176]. Short fibers or particulate reinforcements are inserted into polymer composite FFF to add stiffness, thermal resistance, and multi-functionality [177]. The extrusion direction controls the direction of reinforcement as demonstrated in Figure 11, which leads to strong directional mechanical characteristics. Melt rheology is greatly affected by the incorporation of fillers, which results in high wear rates of nozzles, instability, and sensitivity of interlayer bonding. Therefore, the raster angle, build orientation, layer thickness, and extrusion temperature have a great impact on composite performance [178]. Unlike in conventional molding, the non-uniform thermal history in FFF controls interlayer diffusion and stress development during the process, and process-property interaction is fundamental to structural composite manufacturing [178].

#### 4.1.2. Direct Ink Writing (DIW)

**Figure 12:**
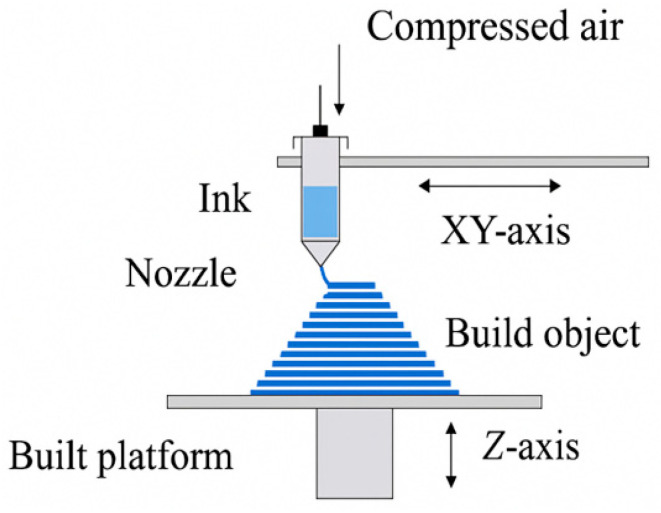
Direct Ink Writing (DIW) process illustration [187].

DIW is an additive manufacturing technique, as shown in Figure 12, whereby viscoelastic composite inks are extruded through a small nozzle under a constant shear stress [180]. Unlike melt-based processes, like FFF, DIW is usually performed at ambient temperatures or slightly higher temperatures, and shape retention during deposition [181] is determined by rheological solidification processes, such as shear thinning, yield-stress behavior, and quick restoration of elastic modulus, and not by temperature reduction [182]. The major strength of DIW is that it can handle highly filled composite systems [183]. These large filler content levels allow making polymer, ceramic, and hybrid-matrix composites that are reinforced by particles, short fibers, whiskers, or nanomaterials [184]. When introducing anisotropic reinforcements during extrusion, the orientation of anisotropic reinforcements can be predetermined due to shear, and the nozzle diameter, extrusion rate, and print path enable the tuning of local stiffness, strength, and anisotropy [185]. This directed orientation is in opposition to the thermally driven bonding processes in FFF and offers more freedom in the design of microstructures. DIW allows the creation of programmable architected composite structures with controllable location of filaments, interlocked porosity, and spatially varying reinforcement orientation. However, it is especially appropriate for functionally graded materials, where gradual changes in composition or microstructure are necessary to control the distribution of stresses or heat. The rheology-controlled deposition, the high versatility of the material, and the ability to tune the microstructure make DIW a potent platform to produce advanced composite parts in an application that needs fine control of the internal architecture and functional performance [186].

### 4.2. Powder-Bed Fusion

#### Selective Laser Sintering (SLS)

**Figure 13:**
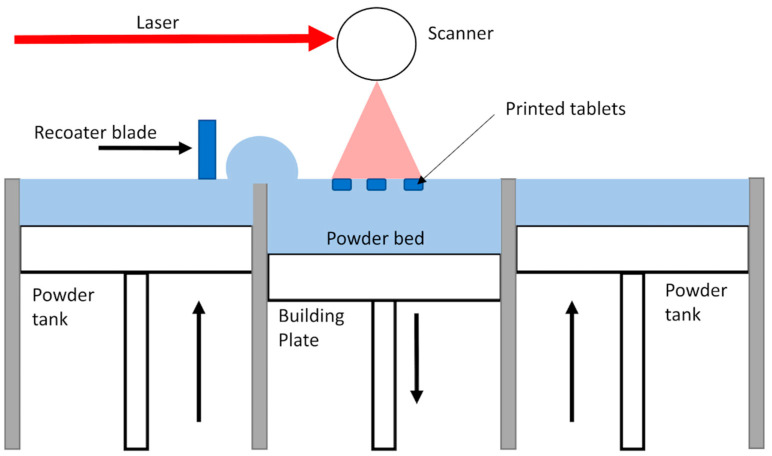
Principle of Selective Laser Sintering (SLS), showing laser scanning, powder deposition by the recoater blade, and layer-by-layer fabrication on the build platform [187].

SLS is a powder-bed fusion process whereby, with a high-energy laser, composite polymer powders are selectively sintered to create solid structures in layers. The presence of surrounding unsintered powder makes any overhangs and complicated geometries easy. It also avoids the need for auxiliary supports and geometric constraints as compared to extrusion methods. In polymer composite SLS, they take the form of reinforcing phases (in short-fiber, ceramic, or nanoscale forms) that are added to thermoplastic powders to increase stiffness, thermal resistance, and dimensional stability. However, several tightly coupled parameters determine composite performance, such as powder morphology, particle size distribution, laser energy density, and scanning strategy. A lack of powder dispersibility or reinforcement dispersion may result in inconsistent densification, porosity, and locally differentiated properties. As shown in Figure 13, neck growth between particles, interlayer bonding, and homogeneity of the microstructure depend on the interaction of laser scanning directions with powder consolidation. The interlayer bonding and anisotropy of SLS are usually more enhanced than extrusion-based AM due to the partial remelting at interlayer interfaces. However, it is important to have accurate thermal control so as to avoid warping, residual stresses, and weakening of reinforcement phases. Therefore, optimization of the composite powder formulations, as well as process-specific laser control strategies, are necessary in terms of obtaining reproducible and high-performance polymer composite parts through SLS [188].

### 4.3. Vat Photopolymerization

#### 4.3.1. Digital Light Processing (DLP)

**Figure 14:**
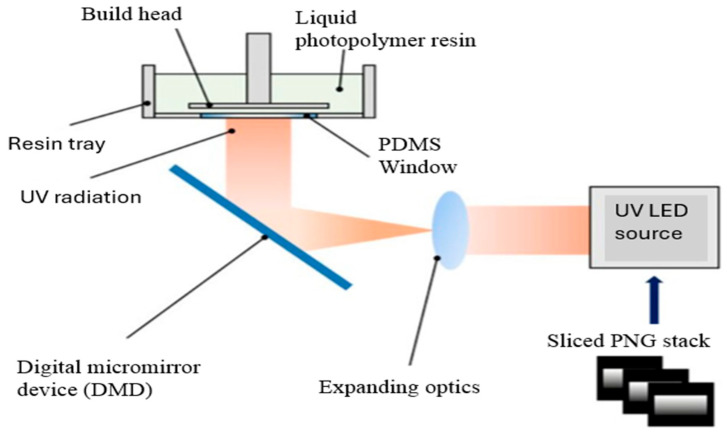
Diagram for Digital Light Processing (DLP) [187].

DLP is a technique of vat photopolymerization where the complete layers of resin are treated in a single cure through the application of spatially modulating UV light, which is reflected through a digital micromirror. Such a layer-by-layer exposure can allow very high in-plane resolution and high building rates, making DLP particularly attractive for the fabrication of polymer nanocomposites with intricate architectures. With carbon nanotubes, graphene, or ceramic nanoparticles, composite DLP systems are implemented with functional fillers, which are dispersed in photocurable resins to deliver required mechanical, electrical, or thermal characteristics. Figure 14 shows that the interaction of the projected UV light with the resin, which contains particles, has a direct effect on cure depth, polymerization kinetics, and feature fidelity. The higher filler level normally results in UV attenuation, light scattering, and an increase in viscosity that may affect the resolution and adhesion of layers unless regulated. Hence, to obtain defect-free composite structures in DLP, a high level of optimization of the exposure energy, resin formulation, and filler surface chemistry is necessary. The recent developments in resin engineering and functionalization of nanoparticles have made DLP shift from prototyping toward the creation of functional composite parts that can be used in electronics, microfluidics, and precision engineering applications [189].

#### 4.3.2. Stereolithography (SLA)

**Figure 15:**
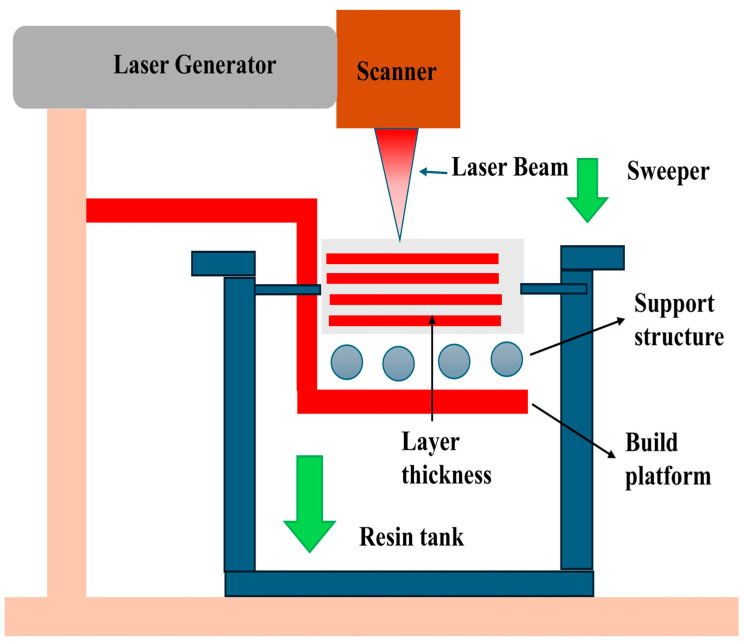
Principle of Stereolithography (SLA), showing laser-induced resin curing and layer-by-layer part formation on the build platform [187].

SLA is a vat photopolymerization process, which is based on point-wise laser exposure or mask-based exposure of liquid photopolymer resins. SLA usually provides better surface finish and dimensional accuracy than DLP, and is therefore well applicable to high-precision composite parts. Functional performance is improved by incorporating nanoscale reinforcers, which include CNTs, graphene, nanoclays, or ceramic particles in polymer composite SLA. Nevertheless, the addition of fillers brings out problems concerning sedimentation, light attenuation, and viscosity regulation. Figure 15 indicates the effect of the light delivery strategy and build platform motion on the cure uniformity and bonding of the layers in the particle-reinforced resins. A lack of proper control may lead to an undue process of curing, or roughness of the surface, or worse, internal defects. New advancements with surface-modified fillers, customized photoinitiator systems, and dynamic resin mixing have made considerable contributions to the expansion of the applicability of SLA in functional polymer composites. These developments make it possible to manufacture high-resolution biomedical product parts, micro-electromechanical systems, and aerospace microstructures, in which materials and dimensional precision are paramount [190].

### 4.4. Droplet-Based Jetting

#### Material Jetting (MJ)

**Figure 16:**
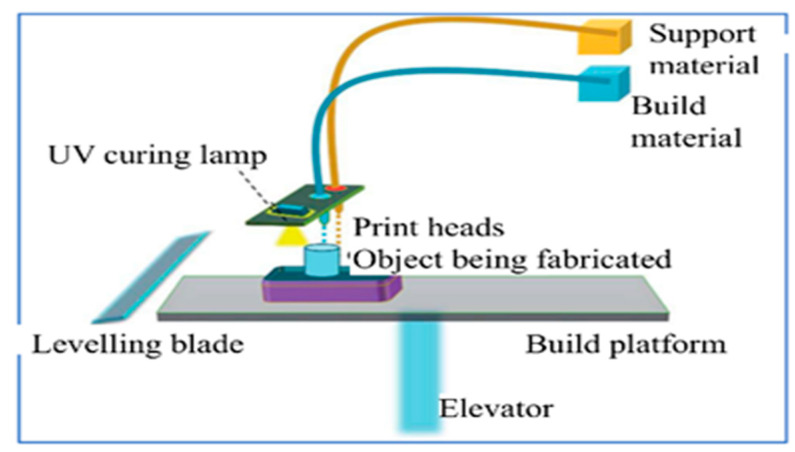
A schematic representation of Material Jetting (MJ) [192].

The droplet-based additive manufacturing processes are based on the deposition of discrete volumes of materials with high positional precision. In MJ, as schematically illustrated in Figure 16, droplets of photopolymers are placed selectively on a build surface, and the droplets are then allowed to cure, providing fine spatial resolution on the location of the materials. MJ is especially beneficial in polymer composites that require local control of properties. The ability to jet multiple materials in a single fabrication makes it possible to prepare heterogeneous composite constructs that can have region-selective mechanical, thermal, or biological functionality. Complex internal channels, overhangs, and graded interfaces can also be achieved by taking advantage of the dissolvable support materials. Composite MJ, however, is limited with regard to ink viscosity, droplet stability, as well as dispersibility of fillers at a micro scale. Future studies are focusing on designing jettable nanocomposite formulations and multi-material printing methods, which retain droplet fidelity with enhanced functional operation [191].

### 4.5. In Situ Curing Strategies

Gao et al. [193] discussed several in situ curing methods that can be united with the DIW process for thermoset printing. These methods include frontal polymerization, electromagnetic heating, photochemical curing, electron beam curing, and resistance heating. Each offers unique advantages for improving print quality and efficiency. For materials such as shape-memory epoxy, a two-stage fabrication method is employed involving UV-assisted printing followed by thermal curing. Photo-curable acrylates first create crosslinked networks during the UV printing stage. Following this, thermal curing polymerizes the epoxy component, resulting in interpenetrating polymer networks (IPNs) that provide significantly enhanced and uniform mechanical properties throughout the material [181]. Dojan et al. [194] explored an innovative manufacturing technique for fiber-reinforced thermoset composites that is both quick and energy-efficient, while also being scalable for larger production. The process uses a thermo-responsive thermoset resin matrix and applies precise remote heating directly to carbon fiber reinforcements. This triggers rapid in situ curing of composite materials during printing, which eliminates the dependency on molds or tooling and phases out the need for any post-printing curing steps.

### 4.6. Multi-Material AM and Functionally Graded AM

**Figure 17:**
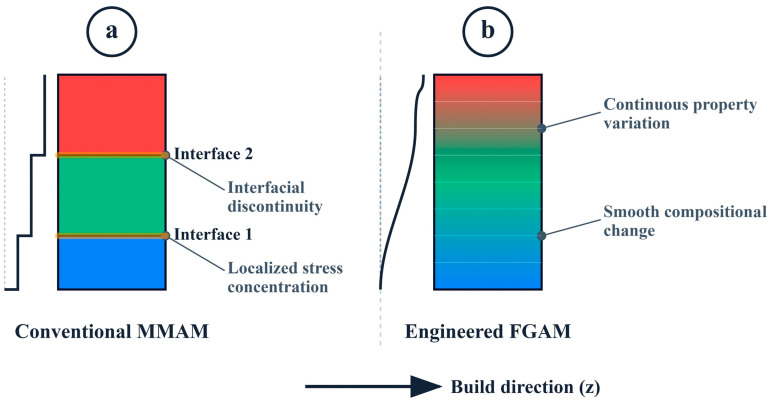
Comparison of (**a**) conventional multi-material additive manufacturing (MMAM) and (**b**) engineered functionally graded additive manufacturing (FGAM) along the build direction (z).

Both emerging Multi-Material Additive Manufacturing (MMAM) and Functionally Graded Additive Manufacturing (FGAM) methods provide a radical departure from geometry-based to performance-based composite design. In comparison to functional graded architectures, the conventional multi-material printing is analyzed, where graded transitions are shown to reduce interfacial stress concentrations and increase the structural integrity. FGAM allows mechanical, thermal, or electrical properties to vary continuously in space within a single component, and is inspired by natural structures such as bone and skin. This is especially useful with applications that need to have a smooth transition of stiffness or localized optimum performance [195]. MMAM also builds on this principle, enabling the selective deposition of different materials in a single build, thereby allowing embedded sense components, conductive traces, and local reinforcement areas. A summary of the properties studied in a multi-material polymer composite AM is shown in Figure 17, with the overwhelming prevalence of mechanical performance, and then by thermal, electrical, and energy-absorption properties. Together, FGAM and MMAM will facilitate the dramatic expansion of the design space of polymer composites in order to permit the use of application-agile architectures and multifunctional components that are not accessible through manufacturing via traditional manufacturing routes [196].

### 4.7. Industrial Adoption and Commercialization Considerations

Although polymer composites have made great progress, AM-fabricated composites tend to have lower mechanical properties (tensile strength and modulus) than traditional processes such as spray-up or pultrusion. In addition, a number of technical problems are still outstanding, including nozzle clogging under high fiber loads, internal porosity, and weakened interlayer bonding (anisotropy), which are being addressed by contemporary research works [33]. The main obstacles in the industry include irregular bonding, dispersion of reinforcement, the emergence of defects, and the inability to scale, which brings about variability, anisotropy, and unreliable long-term behavior to the process of transition to production [130]. This fact is becoming more crucial to the successful industrialization of additively manufactured polymer composites because it is impossible to guarantee reproducibility and consistency of the performance without integrating strict process control with quality assurance frameworks [34]. Although bio-based polymers and natural fiber reinforcements provide the most significant environmental advantages, sustainability-oriented composite systems are limited in their industrial application due to moisture sensitivity, thermal instability, and intrinsic material variability and require better material design and increased manufacturing discipline. In turn, standardized and data-intensive reporting procedures accompanied by quality assurance associated process management and scaled-up break-even demonstrations are a prerequisite for the creation of credible qualification routes and the broadening of motivating high-performance composite AM technologies in the industrial sectors [197,198]. Table 6 summarizes the industrial readiness, scale-up potential, and associated quality assurance (QA) and quality control (QC) considerations of polymer composite AM processes.

## 5. Application Specific Innovations

Additively manufactured polymer composites have expanded their applications across different manufacturing sectors. These materials are lightweight and capable of creating customized complex structures, which support critical needs in the aviation, automotive, marine, healthcare, and electronics sectors.

### 5.1. Aviation Sector

**Figure 18:**
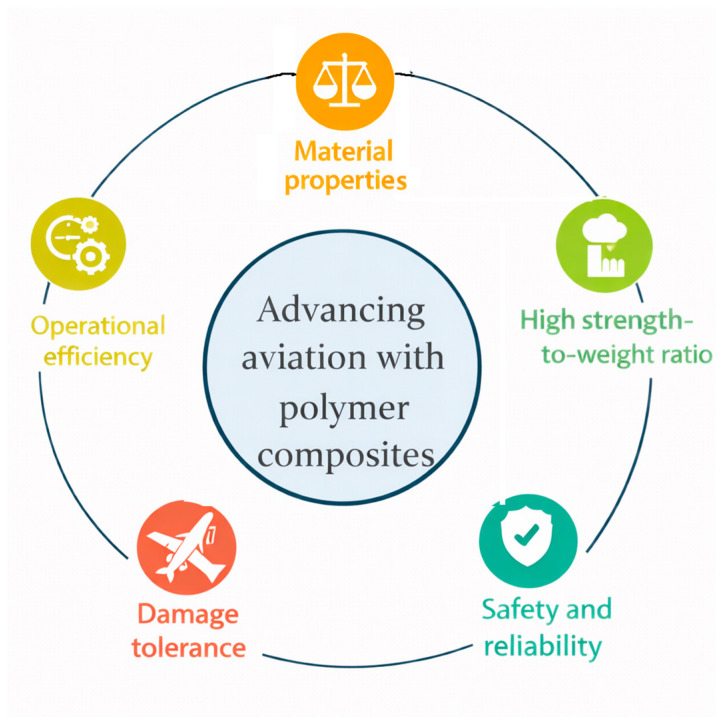
Contribution of polymer composites in materials development and performance enhancement within the aviation sector.

Particularly in the aerospace industry, additively manufactured polymer composites have a major impact on improving aircraft body structure, fuel efficiency, speed, maneuverability, and range. Hybrid fiber–reinforced composites can notably lessen the overall weight of the aircraft in comparison with aluminum alloys. Nanomodified polymer composites provide enhanced radiation shielding to the plane’s body. Carbon fiber-infused composite materials are utilized in the manufacturing of aircraft components subjected to very high-temperature operations [199]. Rahman et al. [198] have reviewed that nanocomposites of polymers with optimized nanoparticle dispersion have much better mechanical, thermal, electrical, and barrier properties than traditional polymer systems. They also concluded that homogeneous dispersion and high interfacial forces are key elements in achieving maximum reinforcement efficiency, as well as multifunctional performance reliability. Consequently, high-performance industries, including aerospace industries, are finding such nanocomposites increasingly appealing. Figure 18 represents the contribution of polymer composites in the aviation sector, including material properties, operational efficiency, safety, damage tolerance, and strength-to-weight performance.

### 5.2. Automobile Sector

Diverse manufacturing techniques are employed to fabricate both small and large automobile parts, including brake pads, body panels, brackets, mounts, engine parts, suspension components, prototypes, and beyond. In particular, metal AM notably cuts down tool manufacturing time while also enhancing vehicle performance [200]. While considering the bumper beam of automobiles, polymer composites, in particular Glass Fiber–Reinforced Polymer (GFRP) composites, are being explored and utilized as a substitute for traditional materials such as cast iron and steel. This alternative results in significant improvements in the bumpers over conventional metals, including higher mechanical strength, weight reduction, and impact damping ability [201]. GFRP composites are capable of reducing weight up to 60% in comparison with bumpers made of steel, without compromising mechanical strength and impact toughness [200]. General applications of GFRP composites in the automobile industry are displayed in Table 7.

### 5.3. Healthcare Sector

**Figure 19:**
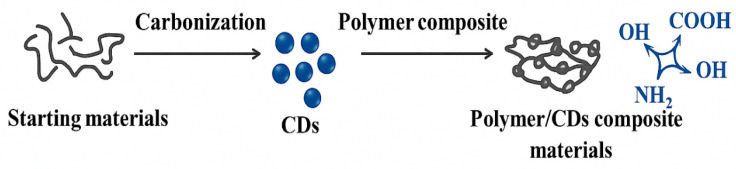
Diagram showing the initial synthesis of CDs from starting materials, followed by the formation of CD/polymer composite structures [204].

To attain the optimal level of mechanical characteristics, electrical conductivity, and essential biocompatibility for applications in medical and biomedical devices, electrically conductive hydrogels (ECHs) are often configured as polymer composites. Usually, these composites consist of conventional insulating polymer matrices. A range of 3D printing techniques (DIW, FFF, SLA) is used on a regular basis to fabricate the polymer composites for ECHs [202]. For a controlled drug and gene delivery system, Carbon dot (CD) based composites are broadly fabricated, notably for chemotherapy drugs: doxorubicin. By integrating insulin into carbon dot/polymer-based hydrogels, smart insulin nanocarriers are made. This ensures the effective delivery of the medicine to the intended tissues [203]. Figure 19 displays the step-by-step synthesis of CDs from raw materials, leading to the creation of CD/polymer composite structures.

### 5.4. Electronics and Energy Sectors

**Figure 20:**
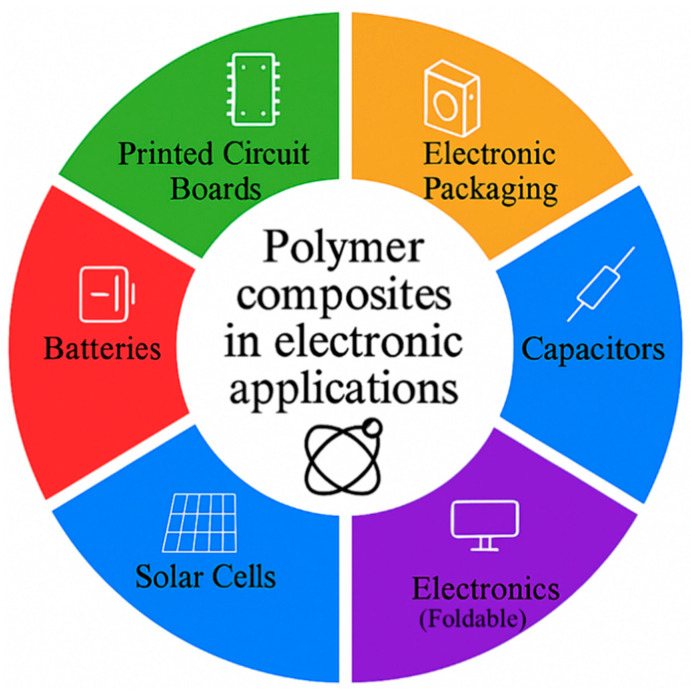
Representation of polymer composites and their functional roles in electronic applications.

Polymer matrix composites are being used in different electronic applications such as PCB board-making, electronic device enclosure applications, passive electronic components, and others. The Printed Circuit Board (PCBs) industry hit $51 billion in 2019 due to the rising demand for electronic products. Glass fiber cloth–reinforced epoxy composites are widely adopted for making PCBs because of their optimal combination of electrical and mechanical characteristics [205,206]. In the context of electronic packaging, 95% of the world’s electronic packaging is made from polymer-based packaging materials, especially thermosets. The reason behind this is affordability, automation suitability, and adaptability. Additionally, epoxy molding compounds tend to be the material of preference for electronic packaging and are effective for use in Plastic Encapsulated Microchips (PEM) [205]. The growing demand for compact, lightweight, and flexible embedded capacitors has propelled research on polymer matrix composites for their use in capacitor components [205]. Polymers possessing an elevated dielectric constant play a crucial role in flexible electronics. Chitosan (CS) and poly(2-ethyl-2-oxazoline) (POZ) materials were combined to fabricate CS: POZ blend films. At a frequency of 1 MHz, these fabricated samples showed a relatively increased dielectric constant value of 6.48 [207]. Graphene-based polymer composites are ideal for manufacturing electronic devices that require frequent folding, bending, and stretching, which traditional electronic devices are unable to facilitate. Few notable applications of graphene-based polymer composites include flexible lighting, displays, flexible energy storage devices, portable electronics, wearable gadgets, and flexible capacitors [208]. Metal oxide-conjugated polymer composites serve as hole-selective/transport layers in perovskite solar cells, increasing their effectiveness and stability [209]. In solid-state batteries, inorganic polymer composites are primarily applied as solid electrolytes. This technique is beneficial for emerging energy storage systems due to several critical features and advantages, including high compactness, suitability for large-scale production, high ionic conductivity, high ion transport number, improved mechanical and chemical stability, and minor safety threat [210]. Figure 20 outlines an overview of polymer composites applications in the electronics field.

### 5.5. Architecture and Construction

Building industries initially utilized FRP composites to reinforce existing structural elements (wooden structures, steel frames, and concrete) or as architectural elements. The first step to construct buildings with composite materials serving as the main load-bearing components was taken in the 1980s. For RC beams, columns, and slabs, FRP composites (natural FRPs and sisal FRPs) act as a dependable solution for reinforcing and structural repairing. Sisal FRP composites play a very effective role in improving the ultimate load-carrying capacity of strengthened RC beams [211,212]. Also, FRP composites provide several vital advantages for bridge construction, such as weight reduction, higher tensile strength, anti-corrosiveness, durability, lower maintenance demands, electro-thermal insulation, and better economic life cycle costing. The first FRP pedestrian bridge was built nearly 50 years ago, and the first road bridge (Ginzi Highway bridge in Bulgaria and Miyun bridge in China) was built in the early 1980s [211]. Rupal et al. [213] investigated an alternative form of concrete that uses a synthetic resin binder rather than the traditional combination of cement and water. This lightweight composite concrete exhibited superior properties, including increased flexural strength, better resistance to chemicals, effective sound insulation, and weight efficiency in comparison to normal concrete. Yang et al. [214] assessed the impact of CNT-enhanced polymer additives on the strength and durability of cement concrete mixtures. The mix was based on Portland cement M500 and included river sand as fillers. Cement paste enhanced with CN gains strength more quickly and becomes noticeably stronger over time than regular mixes. The physical and mechanical properties of cement paste that has been modified using are demonstrated in Table 8.

**Figure 21:**
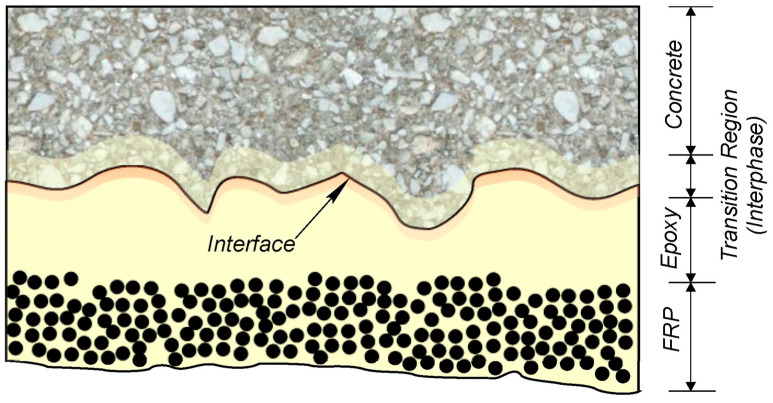
Cross-sectional diagram demonstrating the external bonding of FRP to concrete [216].

Polycarboxylate as a dispersant can greatly enhance the mechanical performance of cement-based nanocomposites. Adding just 0.1% (CNTs) to the polycarboxylate mixture led to a 19.87% boost in compressive strength at 7 days and a 28.65% increase at 28 days. At a higher CNT content of 0.5%, the 28-day compressive strength reached 80.60 MPa, significantly outperforming the 62.66 MPa achieved without polycarboxylate [215]. Nanoclays play a crucial role in minimizing creep deformation in adhesives for FRP—steel bonded joints, allowing the joints to withstand sustained stresses over time with greater stability. In parallel, nanosilica–reinforced epoxy composites show remarkable improvements in bonding strength and resistance to environmental and mechanical degradation, contributing to more robust and long-lasting structural connections [216]. Bonded joints are generally made using concrete, epoxy, and FRP composites, which are shown schematically in Figure 21.

### 5.6. Defense and Sports Sectors

The polymer fiber–reinforced composites are becoming popular in the defense industry because of their high mechanical and thermal performance, low weight, and high resistance to environmental degradation [217]. All these properties allow their common application in defense systems like personal protective equipment, automobile and shipping armor systems, smart defense systems, exterior ballistic panels, and internal cladding systems [218]. Aluminum metal matrix composites (Al-MMCs) are extensively utilized in the making of critical components, which include fins, the fuel tank access panel, rotor blades, and swashplate assemblies of both fixed-wing and rotary-wing aircraft, since they have very good strength-to-weight ratios and thermal stability [219]. They can achieve high weight saving over traditional superalloys without losing strength and creep resistance at extreme conditions [220,221].

Rubber composites have been utilized due to their high elasticity, making them ideal for sports shoes and other protective equipment, in which they are used to dampen shocks and prevent injuries. Sports equipment made using carbon fiber–reinforced composite is designed to improve performance, offers stability in a golf club, and minimizes weight in the bicycle, thereby being more comfortable [222]. The current tennis, badminton, and squash racquets are becoming more and more dependent on advanced composites to boost the velocity of the swings, precision, and general control. They apply these materials in the head and the shafts in order to maximize the energy transfer during a play attempt. Carbon fiber shafts, specifically, offer a higher level of fatigue resistance than steel and can be designed with customized flex profiles, so that players can have repeatable ball flight with a desired final ball trajectory [223,224]. Outsoles are made using a graphene-natural rubber (GNR) composite that is non-weight-bearing and improves tensile strength and fatigue resistance. Better energy rebound and support are offered, and help to minimize foot injuries and lessen leg exhaustion during extended movement [225]. Anti-corrosion paint made out of graphene prevents the erosion of yacht and racing boats’ hulls by seawater. These finishes also decrease the total weight amount, which leads to enhanced performance and speed in high-performance marine use [226].

## 6. Emerging Trends and Research Directions

### 6.1. Digital Design, AI Integration, and Digital Twins

**Figure 22:**
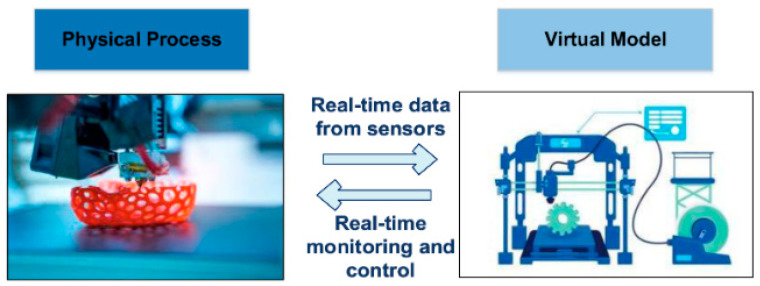
The interaction pathways between the physical and virtual models in the digital twin framework for AM [228].

The addition of digital twins (DTs) to AM is one of the paradigm shifts in the production systems industry under the influence of Industry 4.0. The DTs have been developed to form a network of digital threads, which is a closed one, and share information between the virtual and physical realms of manufacturing in real-time mode, as shown in Figure 22. In this digital continuum, a capability previously unheard of is process monitoring and control, which draws operational data streams via sensor networks to a virtual representation, where the data is continuously compared and tuned. It has been observed that with the relevant implementation of such cyber-physical systems, the rate of defect detection (up to 90% reduction in catastrophic faults) and production rates (30–40 percent reduction in trial runs) have improved in the aerospace and biomedical sectors.

Physical informed neural networks have now allowed defining the evolution of microstructure and formation of defects with computational speeds that are 100–1000 times faster than conventional finite element analysis. Such an approach is seen in the work by Ren et al. [227], who managed to reach the accuracy of 95% in the prediction of the thermal fields in laser-assisted AM with the use of a combined RNN-DNN architecture.

**Figure 23:**
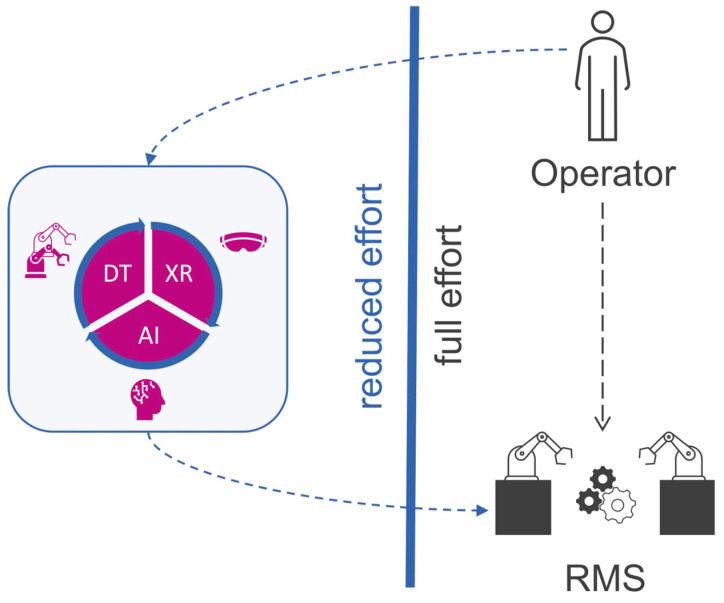
Digital Twin (DT), Extended Reality (XR), and Artificial Intelligence (AI) in Reconfigurable Manufacturing Systems (RMS) [230].

As the interaction of DT with a wider world of extended reality (XR) technologies, as shown in Figure 23, is opening up new boundaries in human–machine collaboration. The latest visualization systems enable the engineer to engage with a virtual prototype in a mixed-reality setting, where design alterations and real-time optimization of the process can be introduced to experience the benefits of the technology. This has led to the slashing of the scaffold development cycle (w/o this integration), which would otherwise take take months or several weeks, and also boosted its mechanical compatibility by 25–35 percent in biomedical applications. Nevertheless, there remain some difficulties in data standardization, model fidelity, and computational infrastructure prerequisites, specifically concerning small-to-medium businesses. Future research directions are concentrated on self-optimizing DTs that implement federated learning and edge computing, which have the potential to empower distributed manufacturing networks, allowing autonomous decision-making [229].

### 6.2. Multi-Material and Hybrid Printing Platforms

Recent studies translate into three important directions of an emerging multi-material polymer composite AM. To start with, the use of AI in process optimization allows real-time modification of parameters based on model-based predictions of melt pool dynamics using convolutional neural networks, whereas more recently, generative design algorithms have been capable of creating structures with spatially varying mechanical properties, resulting in a functionally graded structure. Second, new hybrid systems combine laser-assisted deposition with material extrusion (improving interfacial strength by 92 per cent) and enable multi-nozzle set-ups to print conductive-dielectric composites to accommodate embedded electronics. Three shape memory polymer composites are printed in 4D and have shown the capability to recover a lost shape by 98 percent, and self-healing systems exhibit an 85 percent recovery of mechanical properties [227,231]. Essential issues lie in unifying material interchange procedures and creating in situ curing observation inside hybrid procedures. Short-term work concentrates on bio-inspired elemental structures in terms of hierarchical cellular lattices as impact absorbers (demonstrating 40 percent growth of its stored energy to energy dissipation ratio) and closed-loop material reuse systems. It is being developed into an exploration of fully integrated digital twins with real-time sensor nets, autonomous material selection, and especially soft robotics and energy storage applications [232].

### 6.3. Bio-Inspired and Nature-Mimicking Composites

The idea to use the structure and systems present in nature to formulate advanced materials based on polymer composites also evolved. This has been considered highly advantageous and a scope of promising research. Materials described in case studies are natural fiber-based, such as cellulose and silk, and are of exceptional mechanical strength and toughness, presenting an opportunity for use in aerospace, automotive, and construction industries. In the same manner, the hierarchical composites made of nacre and spider silk displayed brilliant fracture resistance and possessed distinct features of elasticity and biocompatibility, which can be used in biomedical applications and wearable electronics. The use of such composites is varied, and they are extensively used in many industries. The aerospace industry uses such composites to achieve lightweight fuselages, the automotive industry to achieve fuel-efficient bodies and panels in cars, and the biomedical industry applies such composites in tissue engineering and implants. Novel fabrication methods (i.e., AM, electrospinning) allow reproducing the design of natural structures accurately; the novel fabrication methods (i.e., freeze-casting, self-assembly) extend the operational capabilities of the materials. Incorporating bio-inspired concepts and innovative manufacturing, such composites create sustainability and performance that drive engineering across the disciplines [233].

### 6.4. Sustainable and Circular Economy Approaches in Composite Design

With the mass growth of the use of products based on the use of polymers, there have been many problems as well as difficulties that exist ecologically, socially, as well as environmentally and of an economic nature, in turn, leading to a world demand for solutions to sustainable production, through to the waste management facilities. AM or three-dimensional printing (3DP) offers an entirely new production concept that allows a highly efficient, waste-minimized production of functional components with less material requirement and consumption, along with energy expenditure. Its implementation into various fields of industry, such as aerospace, automotive, biomedical, and electronics, bears evidence of its sustainable manufacturing [234]. Through the implementation of circular economy principles, AM can play a role in enhancing the level of sustainability further by enabling the polymeric waste to be recycled and reused through the use of distributed manufacturing systems. With this, the discarded plastics can be converted into quality products, which translates to supporting local production and minimizing environmental effects. The proposed model of a circular economy of AM contains major steps, including material sourcing, creation of filaments, part manufacturing, and recycling them again, guaranteeing the continual flow of the material. The model relies on the focus of decentralized recycling waste plants, fine-tuned feedstock creation, and intelligent design concepts to achieve utmost sustainability. The harmonized work of the researchers, industries, and policymakers is necessary to enhance recycling technologies, enhance material properties, and achieve high penetration of AM towards a circular economy. Through the power of AM, the manufacturing sector and people play an important role in a more sustainable future to eliminate waste and encourage greener production practices [234,235].

### 6.5. Real-Time Monitoring and In Situ Quality Control

The most important novel aspect in AM is the creation of high-quality real-time monitoring and in situ quality control systems, which are making their way as part and parcel of AM as it transitions to full-fledged production. Current developments, like that of the retrofittable monitoring technology (PT 3D) that allows users to perform unceasing dimensional checking and defect sensing in the print stage, illustrate this trend. Those systems take care of some critical issues of industrialization of AM, such as reduction in waste by early failure identification (potentially up to 9–15% materials savings and energy savings in metal AM), improvement of process reliability in situations where failure is not an option, and the generation of valuable data to support material and process development [236].

Such monitoring technologies are especially useful in energy-intensive metal AM processes, which generated 710 million metric tons of Carbon Dioxide Equivalent (MtCO_2_e) emissions in 2020, in view of sustainability. It is projected that these systems have the potential to reduce emissions by 690 MtCO_2_e per year by 2042 by increasing the initial yield rates and scrims. Research in the area is trending towards automated defect identification through integration of artificial intelligence, establishing a standardized set of unitless quality measures, and the design of closed-loop control systems capable of self-calibration of printing parameters. These developments not only correspond to industrializing AM but can also be connected to the general shifts towards smart and sustainable production. The future of this technology will focus on a combination of multi-sensor monitoring systems, standard data protocols, and machine learning algorithms to contribute further to the reliability and repeatability of AM processes across general industrial applications [237].

## 7. Challenges and Limitations

### 7.1. Challenges in Material Formulation and Processing

#### 7.1.1. Poor Interfacial Bonding and Dispersion of Fillers

Even dispersion and good interfacial interactions between the fillers and the polymer matrix are still areas of great challenges and normally undermine the mechanical and functional properties. Although the use of filaments (synthetic or natural) as a reinforcing mechanism of polymers printed using the FFF method can be very promising, there are certain limitations per type. Although synthetic fibers have high mechanical properties in terms of strength and stiffness, they are generally assumed to be more expensive to produce, and they are not classified to be environmentally friendly since they are petrochemical-based products. The fact that they are not biodegradable and renewable means that they are incompatible with sustainable methods [238]. Natural fibers, in their turn, even being renewable, biocompatible, and comparatively low-priced in a great number of cases, show lesser mechanical strength in comparison to their synthetic analogs. They also commonly need pre-treatment so as to promote fiber-matrix adhesion, and they are usually produced in discontinuous form, which restricts their load-bearing capacity. The other disadvantage is that not every natural fiber is readily commercial, and some are even sensitive to ground moisture and degradation. These shortcomings should also be put into serious consideration when choosing fiber type in order to apply functional, cost-effective, and sustainable FFF composites [239].

#### 7.1.2. Printability and Rheological Limitations of High Filler-Content Systems

**Figure 24:**
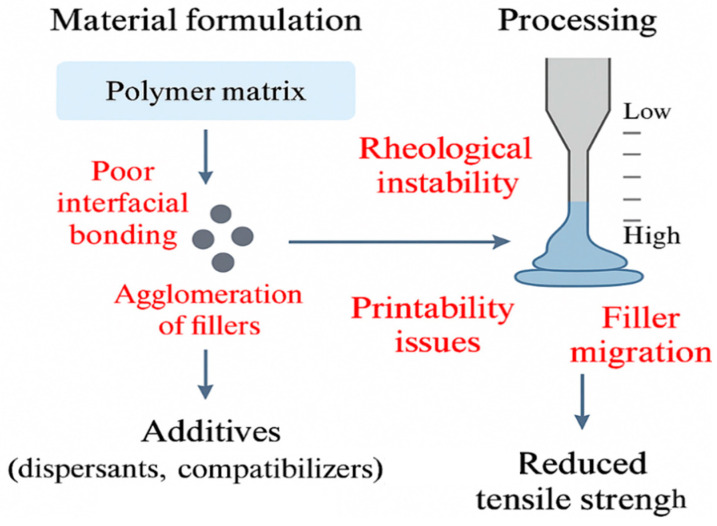
Schematic representation of key challenges in material formulation and processing.

The effect of fillers in high concentrations would have detrimental effects on the flow behavior and printability of the composite and may inhibit the application of the AM solutions. Even though AM technology is continuously evolving, there are also several limitations inherent in existing 3D printing systems. These are low mechanical endurance, low thermal stability, and the life span of 3D-printed objects, in contrast to traditional manufacturing. Being sustainable, however, biomass and recycled polymers cannot necessarily be processed, so in order to increase their structural parameters, additives of macromolecular reinforcing additives are added [240]. The resolution and finish, and speed of printing are also the same, with the challenge of resolution and the surface finish and speed of printing that arises in existing systems, and particularly so in large systems such as the Big Area AM (BAAM). BAAM is much less expensive and material-intensive but has low resolution, poor support continuity and support removal, and insufficient cooling are matched in a reduced amount of printable detail since BAAM has a larger bead [241]. A schematic representation of key challenges in material formulation and processing is shown in Figure 24.

### 7.2. Performance, Scalability, and Sustainability Limitations

**Figure 25:**
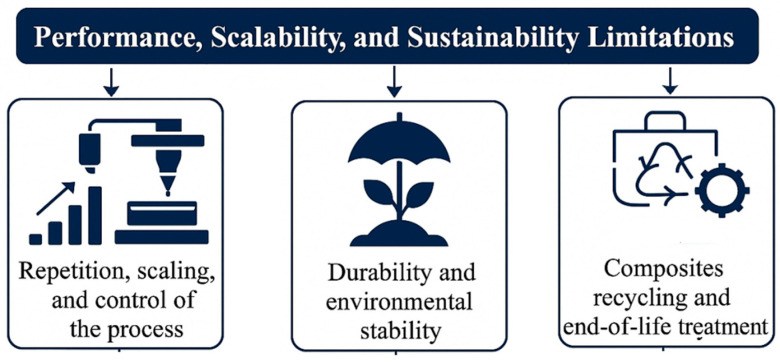
Illustration of performance, scalability, and sustainability limitations of polymer composites for AM.

Polymer composite AM is limited by property variability, scale-up and repeatability issues, and durability under real environments. Sustainability is constrained by energy use, complex treatments, and difficult recycling of heterogeneous composite feedstocks. Figure 25 represents the limitations of polymer composites AM.

#### 7.2.1. Repetition, Scaling, and Control of the Process

To mitigate these limitations, studies frequently employ chemical/physical treatments (alkali, silane, acetylation), compatibilizers (e.g., maleated polymers), hydrophobic coatings, and hybrid reinforcement strategies (natural–synthetic fiber blends or nano-additives). However, such treatments increase cost and processing complexity and may partially offset sustainability gains. Therefore, natural-fiber AM composites should be positioned as high-value sustainable solutions primarily for semi-structural components, interior parts, and moderate-load applications unless durability-enhancing strategies and long-term aging evidence are demonstrated. There are serious technical challenges in creating similar quality throughout the batches, as well as in scaling up production from laboratory to industrial scales [242]. MMAM can be used to manufacture spatially variable properties parts, but is somewhat challenged in terms of material compatibility, residual stresses, and poor interfacial bonding. Cracks, warping, and poor adhesion could be caused by thermal and mechanical differences between materials. This field of research is relatively young and still not much validated with finite element modeling, slicing, and design software are also underdeveloped [243]. In order to develop the MMAM, it is becoming more important to have better material selection, enhanced process control, and wiser software tools.

#### 7.2.2. Durability and Environmental Stability

Composite performance can be lost to other environmental conditions, especially when implemented in harsh or outdoor applications. Natural fiber reinforced composites are more prone to moisture absorption, UV exposure, and microbial attack, two factors that can cause premature deterioration. Although synthetic composites are more resistant, they have some problems, such as a mismatch in thermal expansion and creep due to sustained loads [244]. Enhanced testing to determine the susceptibility of AM polymer composites to accelerated aging and protective coatings is under investigation, but long-term performance data will be needed on many specific composites.

#### 7.2.3. Composites Recycling and End-of-Life Treatment

Recycling and environmentally feasible disposal are issues because fiber-reinforced or nanoparticle-filled systems are heterogeneous and therefore pose an environmental concern. The existing restrictions on the ecological efficiency of the majority of AM processes are caused by high power consumption, the application of dirty emissions, and the non-existence of efficient recycling operations [244]. Mechanical recycling tends to cause degradation and breakage of fibers and depreciation of properties, whereas chemical recycling methods are still energy-consuming and expensive. New systems may present more sustainable solutions, such as closed-loop recycling systems and bio-based, biodegradable composites, but their investment is impractical because profitability is highly dependent on economics and technology [245]. Table 9 provides a structured snapshot of chief bottlenecks in composite-based AM, grouping concerns like filler dispersion, mismatched material properties, equipment constraints, limited scalability, and unresolved end-of-life pathways.

## 8. Conclusions

Polymer composites are quietly changing the landscape of AM, especially in areas like aerospace, biomedical devices, and electronics, where lightweight and multifunctional parts really matter. By blending polymers with reinforcements such as carbon fibers, graphene, CNTs, or even natural fibers, researchers have managed to enhance properties such as strength, thermal stability, and conductivity, at least in many cases. But the outcomes are not always consistent; a lot depends on how well the fillers are dispersed or how strong the bonding is at the interface. On the process side, newer AM methods such as FFF, SLA, DLP, DIW, and multi-material printing are opening the door to more complex designs and custom-tailored properties, though not without their own limitations. There is also growing excitement around ideas like 4D printing, smart materials, or composites that can self-heal or respond to stimuli, but these are still emerging and probably not ready for broad industrial use just yet. Meanwhile, the shift toward sustainable or bio-based composites is picking up speed, although matching their performance to conventional materials remains a work in progress. Some challenges, such as scaling up production, ensuring long-term durability, or improving process repeatability, remain persistent obstacles. Moving forward, real progress will likely require not just better materials, but smarter integration between processing, design, and maybe even machine learning to guide it all.

## Figures and Tables

**Table 1:** Summary of the major printing parameters used for fabricating the 3D printed structures [50].

Sample	Nylon 6,6	Nylon 6,6/30%CF
Printing temperature (°C)	270	360
Bed temperature (°C)	60	80
Printing speed (mm/s)	15	15
Layer thickness (mm)	0.2	0.2
Infill (%)	100	100
Infill pattern	Rectilinear	Rectilinear
Nozzle diameter (mm)	0.5 mm	0.5 mm
Material flow rate multiplier	1.0	1.5

**Table 2:** Mechanical properties of natural fibers [74].

Natural Fiber	wt.% Loading	Matrix Material	Tensile Strength (MPa)	Flexural Strength (MPa)	Impact Strength	References
Kenaf	30–40	PLA	50–61	58–62	15–48 kJ/m^2^	[75]
	40	PP	90	50	-	[76]
	30	PLA	36.18	64.90	116.6 J/m	
Jute	50	Epoxy	39.52	89.62	2.22 J	[77,78]
	26.9	Epoxy	70.4	84	-	[78]
	33	PP	27.49	43.33	25.54 kJ/m^2^	[79]
Flax	37.9	Epoxy	95.4	95	-	[78,80]
	20	PLA/PCL (70:30)	49–60	-	3.3–6 kJ/m^2^	[81]
	22	PLA	-	160–185	-	[82]
Hemp	50	Epoxy	22.43	57.11	1.25 J	[77,83]
	30–50	PLA	39–65	51–113	-	[84]
	30	Polybenzoxazine	52	122	4.23 kJ/m^2^	[85]
Abaca	10–30	PP	22–30	46–54	0.040–0.048 kJ/m^2^	[86]
	20–50	BioPE	26.64–47.73	-	-	[86]
	30	HDPE	33.13	-	-	[87]
Coir	10–30	PP	24–30	48–57	0.040–0.055 kJ/m^2^	[88]
		Melamine-Urea-				
	84–90	Formaldehyde (MUF)	3.05–4.4	2.099–5.149	-	[89]
		Biopolymer				
	5–30	Bakelite resin	-	53–61	-	[90]

**Table 3:** The Mooney viscosity values of the selected rubber materials, as specified by the manufacturer [111].

	Natural Rubber (NR)	Ethylene-Propylene-Diene Rubber (EPDM)	Nitrile-Butadiene Rubber (NBR)
Mooney viscosity (ML1 + 4; 100 °C)	57	112	37
Vulcanization Conditions Dumbbell specimen S2 (10 min)	160 °C	170 °C	170 °C

**Table 4:** Functioning methods and mechanisms of SMPs [143].

Method of Activation		Functioning Mechanism
By heating	Direct heating	Warming the polymer above its transition temperature
	Indirect heating	Activated by means of joule heating through the application of electrical voltage
	Induction heating	Activation of the SMPs via eddy currents induced using an alternating electromagnetic field
By solvents	Water	By acting as a plasticizer, the solvent increases the movement of polymer chains, which in turn shortens relaxation time and lowers the glass transition temperature.
	Solvent	
By light	Photo-reversible cycloaddition reactions	SMPs can effectively undergo photo-reversible cycloaddition reactions in response to specific wavelengths of light
	Photo-thermal effect	By incorporating photo-thermal fillers into the SMP matrix, electromagnetic radiation can be converted into heat.

**Table 5:** Key chemical constituents and structural components forming the geopolymer network [170].

Constituents	Composition	Temperature [°C]	Chemicals	Compressive Strength [MPa]
Coal fly ash and metakaolin	-	250	NaOH	-
Fly ash, GGBFS, and zeolite	Al/Si	32	NaOH	100
Coal fly ash	Al/Si	80	NaOH	18
Fly ash	SiO_2_/Al_2_O_3_	85	KOH	19

**Table 6:** Comparative assessment of polymer composite AM techniques with respect to industrial readiness and scale-up [33,198,199,200].

AM Process Family	Composite Feedstock Readiness	Industrial Strengths	Key Limitations	QA/QC Scale-Up Focus	Typical Best Use
Material extrusion (FFF, DIW)	High	Low cost, flexible	Anisotropy, clogging	Moisture, bead control	Prototypes, fixtures
Vat photopolymerization (DLP, SLA)	Moderate	High resolution	Scattering, brittleness	Cure depth, resin stability	Precision parts
Polymer PBF (SLS)	Moderate–High	Support-free, complex	Porosity, powder variation	Powder size, energy density	Batch components
Material jetting (MJ)	Low–Moderate	Multi-material accuracy	Limited fillers, cost	Droplet control, curing	Multi-material prototypes
Continuous fiber AM (CFR-AM)	Low–Moderate	Very high strength	Fiber steering limits	Fiber alignment, bonding	Structural parts

**Table 7:** Common uses of GFRP components in modern automotive design [200].

Automobile Modules	Manufacturing Company	Key Findings
Leaf spring	Chevrolet Corvette C4	15 kg weight reduction
Suspension spring	Audi AG	Achieves a 40% lower weight than its steel equivalent
Instrument and indoor panel modules	Landover Evoque	-
Door module	Faurecia Jeep Liberty SUV	-
Fluid filter module	Daimler AGT–Mercedes	-
Bumper beam	-	Improved ability to absorb shocks without damage

**Table 8:** Physical and mechanical properties of CN modified cement paste [214].

		Compressive Strength, MPa		
		Time Duration in Days		
Carbon Nanotube Content, Mass %	Normal Density %	1	7	28
0.1	26.0	23.7	32.8	60.8
0.5	25.6	21.2	30.4	62.9

**Table 9:** Major challenges in polymer composite AM-material formulation, processing, durability, and sustainability.

Challenge	Description
Filler Dispersion and Bonding	Weak interactions leading to reduced mechanical strength.
Fiber Trade-offs	Balancing the properties of synthetic and natural fibers.
High Filler Content	Causes poor flow behavior, clogging, and reduced structural integrity.
System Limitations	Issues like poor resolution, clogging, and slow processing speeds.
Repetition and Scale-up	Inconsistent quality and performance across production batches.
Software and Design Gaps	Limited modeling, slicing, and simulation capabilities.
Durability and Exposure	Degradation from UV radiation, moisture, and microbial attack.
Recycling Barriers	Inefficient recycling due to material heterogeneity.

## Data Availability

No new data were created or analyzed in this study.
